# Studying the influence of external moment and force on a disc’s motion

**DOI:** 10.1038/s41598-022-21199-z

**Published:** 2022-10-09

**Authors:** T. S. Amer, W. S. Amer, H. El-Kafly

**Affiliations:** 1 Mathematics Department, Faculty of Science, Tanta University, Tanta, 31527 Egypt; 2 Department of Mathematics and Computer Science, Faculty of Science, Menoufia University, Shebin El-Kom, 32511 Egypt; 3 Tanta Higher Institute of Engineering and Technology, Tanta, Egypt

**Keywords:** Applied mathematics, Mathematics and computing

## Abstract

In this work, the influence of a gyrostatic moment vector (GMV) and the Newtonian field (NF) on the rotatory motion of a restricted rigid body (RB) according to disc case around a fixed point is examined. The basic equation of the body motion is used to get the regulating motion’s system as well as the three available independent first integrals. The system’s six equations and these integrals were reduced to two equations of a quasi-linear two-degrees-of-freedom autonomous system and one first integral. The disc has been presumed to be quickly rotating around one of the ellipsoid of inertia's main axis. Poincaré’s method of small parameter (PMSP) is applied to acquire the periodic solutions of the controlling system of the body’s motion. Euler's angles are utilized to characterize the body’s configuration at any instant in which it is graphed, as well as the obtained solutions to explore the good action of the body’s parameters on its motion. The phase plane graphs of these solutions are presented to examine their stabilities. The relevance of this work may be traced to its wide range of applications in fields as diverse as physics, engineering, and life sciences, including assembly and machine design.

## Introduction

The rotational motion of a RB problem around a fixed point in different fields such as gravitational, Newtonian, and electromagnetic fields is regarded as one of the most difficult mathematical problems in mechanics. The difficulty of this problem is due to the fact that it is controlled by a system of six nonlinear differential equations and three first integrals^1^. The exact solutions of these equations demand another adoption fourth first integral. With the exception of a few particular circumstances including Euler–Poinsot, Lagrange–Poisson, and Kovalevskaya, numerous trials have been carried out to find this integral in its entire generality^2^. A large number of integrable cases for the RB’s motion were obtained in^3–5^.

Since, it is difficult or even nearly impossible, until now, to get the general solutions of this problem because the fourth prime integral is not achieved in its general form. It may require looking at the perturbation methods^6,7^ to gain the approximate solutions of this problem.

The nonlinear vibrational motion of the RB in a plane is examined in several scientific works, e. g.^8–10^. In^8^, the authors considered the case of a fixed pivot point and three cases of resonance were examined simultaneously in the context of the approach of multiple scales^7^. The cases of moving suspension point of the damped RB pendulum with three-degrees-of-freedom (DOF) were investigated in^9,10^. The AMS was applied to obtain the analytic solutions of the controlling systems and the stability regions were determined and analyzed.

The averaging method^7,11^ has a wide spread in many scientific works to address the rotational motion of the RB problem, whether in the symmetric gravitational field^12,13^ or the NF^14,15^ or even in the presence of a gyrostatic moment and a magnetic field^16–18^. These works were explored when the applied operating perturbing moments along the primary axes of inertia are assumed to be constants. A tiny parameter is inserted into the regulating systems using some of the initial circumstances to gain the corresponding averaging system. The latter system is developed and numerically solved for a scenario analogous to Lagrange's gyroscope in^19^, in which the applied moments are expressed in terms of the angular velocity projections on the main axes. The case of the variation of these moments with time was examined in^20^. In^21^, the author provided another generalization of this problem when the body is acted by external moments and forces. Further details on how AM can be used to solve the RB problems can be found in^11^.

The PMSP was used in^22,23^ to acquire the solutions of the controlling system of the RB’s motion in a constant gravitational field. When the influence of NF and the GMV are taken into consideration as in^24–27^, a generalisation of this problem is provided. It is noted that the obtained solutions in^22–24^ have points of singularity, while the achieved ones in^25–27^ don’t have any singularities at all. These singularities appear when the natural frequency of the controlling system of motion matches with the integer values or its multiple inverses. In addition, when the body’s center of mass is shifted a little away from the body’s axis of dynamics symmetry, the same method was used in^28^ to gain the solutions of the regulating equations of motion (EOM) for a symmetric RB when the gravitational field is acted. This problem is highlighted in^29^ when the projections of the GMV on the body's main axes are considered. More investigation of this problem can be found in^30–32^ when the RB’s motion was influenced by the NF, GMV, and electromagnetic field, respectively. The influenced spinning motion of a RB by NF was examined in^33^ as a special case of a disc, in which the gained solutions have singular points. These singularities have been separately treated according to the values of the body’s natural frequency in^23,34^. The impact of one component of the GMV on the disc’s motion is investigated in^35^. The importance of this component lies in the fact that the solutions that have been reached do not contain any singular points. It has contributed to achieve a new frequency called the Amer’s frequency^26,27,36^, which in turn showed that there are no singularities in the solutions of RB’s motion at all.

The influence of the GMV and NF on the rotatory motion of a RB analogous to the disc case is investigated in this paper. The EOM are obtained using the basic equation of body motion and the three possible independent first integrals are gained. These equations, as well as the integrals, are condensed into just two quasi-linear 2DOF autonomous system and one first integral. The disc is supposed to be rapidly rotating around one of the main axes of the ellipsoid of inertia. In light of this system, the periodic solutions of the EOM are obtained using the PMSP, in which they don’t have any singularities at any value of the disc’s frequency. Euler's angles are employed to describe the body's current configuration and they are graphed as well as the computed solutions to reveal the positive effect of the body's parameters on its motion. The phase plane of the acquired solutions are plotted to assess their stability. The rest of the paper is organized as follows: “Problem’s formulation” is devoted to describe in detail the examined problem and to formulate the EOM besides the corresponding first integrals. In “Reduction of the controlling system”, the governing EOM and their integrals are reduced of a system of two quasi-linear differential equations and one integral. “Constructing the periodic solutions” presents the desired periodic solutions applying the PMSP for a positive value of the system’s frequency. Euler’s angles are examined in “Dynamical analysis of the body’s motion” to determine the orientation of the RB at any instant. The graphical depictions of the obtained outcomes are examined and discussed in “Outcomes analysis”. A conclusion of the achieved results is given at the end of this work.

## Problem’s formulation

**Figure 1:**
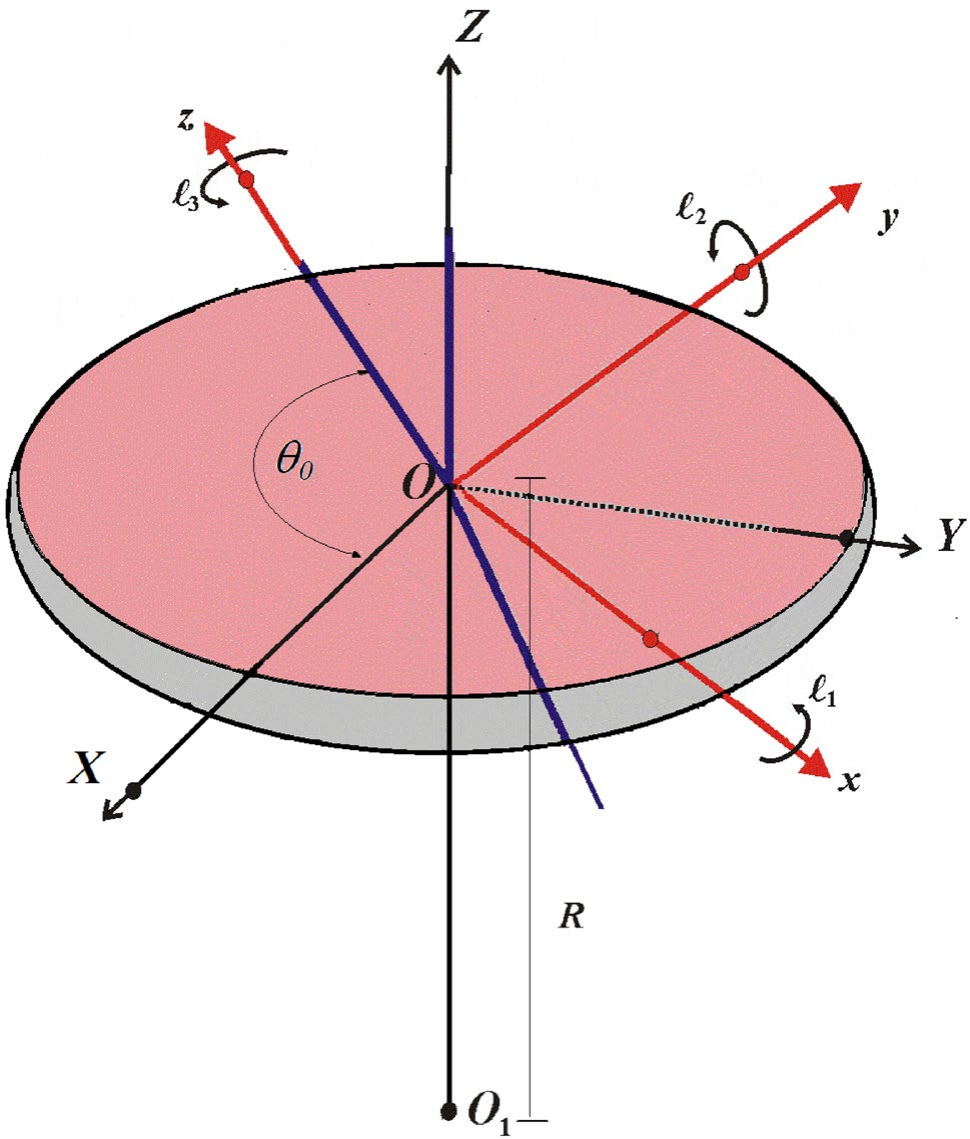
The force component.

Let us consider a disc of mass and fixed point $M$ and $O$, respectively. Two frames of Cartesian coordinates are considered, in which they have the same origin $O$; the first is fixed frame in space $XYZ$, and the other is attached to the disc and moving with it. The disc’s motion is influenced by the NF which is developed from an attraction center $O_{1}$ at a distance $R = OO_{1}$ and the GMV $\underline{\ell }$ whose projections $\ell_{j} \,(j = 1,2,3)$ are directed along the principal axes $x,y,z$ of ellipsoid of inertia, see (Fig. 1). To proceed with the problem’s description, we suppose that the body revolves with a high angular velocity $r_{0}$ around the $z$-axis, which generates an angle $\theta_{0} \approx \pi /2$. As a result, the equations of Euler-Poisson that characterise the body’s motion can be deduced from the fundamental angular momentum's equation $\underline{{\dot{h}}}_{o} + \underline{\omega } \wedge \underline{{h_{0} }} = \underline{G}_{o}$ and the directional cosine equations^1^; where $\underline{{\dot{h}}}_{o}$ denotes the variation of the angular momentum vector $\underline{h}_{o} = (Ap + \ell_{1} ,Bq + \ell_{2} ,Cr + \ell_{3} )$ regarding time at $O$, $\underline{G}_{o}$ represents the instantaneous moment vector of the external forces regarding to the same place, $(p,q,r)$ are the components of the vector of angular velocity $\underline{\omega }$ on the main axes of inertia ellipsoid, and $(A,B,C)$ are the values of these axes. Therefore, the basic dynamic equations for the RB have the form^37^

1 $$\begin{aligned} & A \frac{{dp}}{{dt}} + A q r - r\ell _{2} + q \ell _{3} = Mgy_{0} \gamma ^{\prime\prime} + NA\gamma {^{\prime}}\gamma ^{\prime \prime}, \\ & B \frac{{dq}}{{dt}} - B rp \, - p\ell _{3} + r\ell _{1} = - Mgx_{0} \gamma ^{\prime \prime}\, - NB\gamma ^{\prime \prime}\,\gamma , \\ & C \frac{{dr}}{{dt}} - (\,A - B\,) p q + p \ell _{2} - q \ell _{1} = Mg(x_{0} \gamma {^{\prime}} - y_{0} \,\gamma )\, - N(A - B)\,\gamma \,\gamma{^{\prime}}, \\ & \frac{{d\gamma }}{{dt}} - r\,\gamma {^{\prime}} + q \gamma ^{\prime \prime} = 0,\,\,\,\,\,\frac{{d\gamma {^{\prime}}}}{{dt}} - p\,\gamma ^{\prime \prime} + r \gamma = 0,\,\,\,\,\,\,\frac{{d\gamma ^{\prime \prime}}}{{dt}} - q\,\gamma + p \gamma {^{\prime}} = 0; \\ & N = 3g/R,\quad g = \lambda /R^{2} . \\ \end{aligned}$$

where $$(x_{0},y_{0},0)$$ represent the center of mass’s coordinates in the rotating frame, $$(\gamma,\gamma{^{\prime}},\gamma^{\prime \prime})$$ are the projections of the unite vector $$\underline{{\overset{\lower0.5em\hbox{$\smash{\scriptscriptstyle\frown}$}}{K} }}$$ that located along *Z*-axis, $$g$$ is the acceleration due to gravity, and $$\lambda$$ is the attracting center coefficient.

It's essential to mention that system of Eq. (1) has the below integrals of energy, area, and the geometric integral

2 $$\begin{aligned} & Ap^{2} + B q^{2} + Cr^{2} - 2Mg(x_{0} \gamma + y_{0} \gamma{{^{\prime}}}) - N(A\gamma{^{{\prime} 2}} + B\gamma^{2} ) = Ap_{0}^{2} + Bq_{0}^{2} + Cr_{0}^{2} - 2Mg(x_{0} \gamma_{0} + y_{0} \gamma{^{{\prime}}_{0}} ) - N(A\gamma{^{{\prime}}_{0}}^{2} + B\gamma_{0}^{2} ), \\ & (Ap + \ell_{1} )\gamma + (Bq + \ell_{2} )\gamma{{^{\prime}}} + (Cr + \ell_{3} )\gamma^{\prime \prime} = (Ap_{0} + \ell_{1} )\gamma_{0} + (Bq_{0} + \ell_{2} )\gamma^{{\prime}_{0}} + (Cr_{0} + \ell_{3} )\gamma{^{{\prime\prime}}_{0}} , \\ & \gamma^{2} + \gamma{^{{\prime 2}}} + \gamma{^{{\prime \prime 2}}} = 1, \\ \end{aligned}$$

where $(p_{0},q_{0},r_{0} )$ and $(\gamma_{0},\gamma^{\prime}_{0},\gamma^{\prime \prime}_{{0}})$ denote the values of $(p,q,r)$ and $(\gamma,\gamma{^{\prime}},\gamma^{\prime \prime})$ at $t = 0$, respectively.

We will now examine the disc’s case, i.e. ($A + B = C$) and consider the below variables and parameters

3 $$\begin{aligned} & p = c\sqrt {\gamma{^{{\prime\prime}}_{0}} } p_{1} ,\quad q = c\sqrt {\gamma{^{{\prime\prime}}_{0}} } q_{1} ,\quad r = r_{0} r_{1} ,\quad k = N/c^{2} \quad \quad (\;. \equiv d/d\tau ), \\ & \gamma = \gamma{^{{\prime\prime}}_{0}} \gamma_{1} ,\quad \gamma{^{\prime}} = \gamma{^{{\prime\prime}}_{0}} \gamma^{{\prime}_{1}} ,\quad \gamma^{\prime \prime} = \gamma{^{{\prime\prime}}_{0}} \gamma{^{{\prime\prime}}_{1}} ,\quad t = \tau /r_{0} ,\quad \gamma_{0} > 0,\quad 0 < \gamma{^{{\prime\prime}}_{0}} < 1; \\ & \varepsilon = c\sqrt {\gamma{^{{\prime\prime}}_{0}} } /r_{0} ,\quad C_{1} = (B - A )/C,\quad a = A/C,\quad b = B/C,\quad a + b = 1,\quad \\ & c^{2} = Mgl/C,\quad x_{0} = lx^{\prime}_{0} ,\quad y_{0} = ly^{\prime}_{0} ,\quad l^{2} = x_{0}^{2} + y_{0}^{2} , \\ \end{aligned}$$

where $0 < \varepsilon < < 1$ refers to a small parameter.

Based on the foregoing, the controlling system (1) and its integrals (2) have the following forms:

4 $$\begin{aligned} & \dot{p}_{1} + q_{1} \,r_{1} + A^{ - 1} [\,r_{0}^{ - 1} q_{1} \,\ell_{3} - (c\sqrt {\gamma{^{{\prime\prime}}_{0}} } \,)^{ - 1} \,r_{1} \ell_{2} \,] = \varepsilon \,a^{ - 1} \,(\,y^{\prime}_{0} \,\gamma{^{{\prime\prime}}_{1}} + k\,a\,\,\gamma{^{{\prime}}_{1}} \,\gamma{^{{\prime\prime}}_{1}} \,\,), \\ & \dot{q}_{1} - \,p_{1} \,r_{1} - B^{ - 1} [\,r_{0}^{ - 1} p_{1} \,\ell_{3} - (c\sqrt {\gamma{^{{\prime\prime}}_{0}} } \,)^{ - 1} \,r_{1} \ell_{1} \,] = - \varepsilon \,b^{ - 1} \,(x^{\prime}_{0} \,\gamma{^{{\prime\prime}}_{1}} + k\,b\,\,\gamma_{1} \,\gamma{^{{\prime\prime}}_{1}} \,\,), \\ & \dot{r}_{1} = \varepsilon^{2} \,[\;(c\,C\,\sqrt {\gamma{^{{\prime\prime}}_{0}} } \,)^{ - 1} \,\,(\,q_{1} \ell_{1} - p_{1} \ell_{2} \,) + (\,x^{\prime}_{0} \,\gamma^{\prime}_{1} - y^{\prime}_{0} \,\gamma_{1} - C_{1} \,p_{1} \,q_{1} + k\,C_{1} \,\gamma_{1} \,\gamma^{\prime}_{1} )\;], \\ & \dot{\gamma }_{1} = r_{1} \,\gamma^{\prime}_{1} - \varepsilon \,q_{1} \,\gamma{^{{\prime\prime}}_{1}} ,\quad \quad \dot{\gamma }^{\prime}_{1} = \varepsilon \,p_{1} \,\gamma{^{{\prime\prime}}_{1}} - r_{1} \,\gamma_{1} ,\quad \quad \dot{\gamma }{^{{\prime\prime}}_{1}} = \varepsilon \,(\,q_{1} \,\gamma_{1} - p_{1} \,\gamma^{\prime}_{1} \,)\,; \\ \end{aligned}$$

5 $$r_{1}^{2} = 1 + \varepsilon^{2} S_{1} ,\quad r_{1} \gamma{^{{\prime\prime}}_{1}} = 1 + \varepsilon S_{2} ,\quad \gamma_{1}^{2} + \gamma{^{{\prime}}_{1}}^{2} + \gamma{^{\prime\prime}}{_{1}}^{2} = (\gamma{^{{\prime\prime}}_{0}} )^{ - 2} ;$$

where

6 $$\begin{gathered} S_{1} = a(p_{{10}}^{2} - p_{1}^{2} ) + b(q_{{10}}^{2} - q_{1}^{2} ) - 2[x_{0}{^{\prime }} (\gamma _{{10}} - \gamma _{1} ) + y_{0}{^{\prime }} (\gamma _{10}^{\prime} - \gamma _{1}{^{\prime }} )] + k[a(\gamma _{{10}}^{2} - \gamma _{1}^{2} ) + b(\gamma _{{10}}{^{{\prime 2}}} - \gamma _{1}{^{{\prime 2}}} ) + (1 - \gamma _{1}{^{{\prime \prime 2}}})], \hfill \\ S_{2} = a(p_{{10}} \gamma _{{10}} - p_{1} \gamma _{1} ) + b(q_{{10}} \gamma _{{10}}{^{\prime } } - q_{1} \gamma _{1}{^{\prime }} ) + (cC\sqrt {\gamma _{0}{^{{\prime \prime }}}})^{{ - 1}} [\ell _{1} (\gamma _{{10}} - \gamma _{1} ) + \;\ell _{2} (\gamma _{{10}}{^{\prime }} - \gamma _{1}{^{\prime }} ) + \ell _{3} (1 - \gamma _{1}{^{{\prime \prime }}} )]. \hfill \\ \end{gathered}$$

## Reduction of the controlling system

This section's goal is to reduce system (5) and integrals (6) into a more manageable system comprised of two second-order differential equations and a single integral. To fulfil this goal, one may represent the variables $r_{1}$ and $\gamma{^{{\prime\prime}}_{1}}$ through the below forms using the early two equations of (5).

7 $$\begin{aligned} & r_{1} = 1 + \frac{1}{2}\varepsilon^{2} [S_{1} - k(1 - \gamma{^{{\prime\prime}}_{1}}^{2} )] + \cdots , \\ & \gamma{^{{\prime\prime}}_{1}} = 1 + \varepsilon S_{2} - \frac{1}{2}\varepsilon^{2} [S_{1} - k(1 - \gamma{^{{\prime\prime}}_{1}}^{2})] + \cdots . \\ \end{aligned}$$

Differentiate the first equation of system (4) to get

$$\ddot{p}_{1} + (\dot{q}_{1} \,r_{1} + q_{1} \,\dot{r}_{1} ) + A^{ - 1} [r_{0}^{ - 1} \dot{q}_{1} \,\ell_{3} - (c\sqrt {\gamma{^{{\prime\prime}}_{0}} } )^{ - 1} \,\dot{r}_{1} \ell_{2} \,] = \varepsilon \,a^{ - 1} [y^{\prime}_{0} \,\dot{\gamma^{\prime \prime}}_{1} + ka(\dot{\gamma_{1}^{\prime}} \gamma_{1}^{\prime\prime} + \gamma^{\prime}_{1} \dot{\gamma^{\prime \prime}}_{1} )].$$

Substituting $\dot{r}_{1},\dot{\gamma{^{\prime}}}_{1},\dot{q}_{1},$ and $\dot{\gamma^{\prime \prime}}_{1}$ from (4) into the previous equation to obtain

$$\begin{aligned} & \ddot{p}_{1} + [ - \varepsilon b^{-1} (x^{\prime}_{0} \gamma{^{{\prime\prime}}_{1}} + k\,b\,\gamma_{1} \gamma{^{{\prime\prime}}_{1}} ) + \,p_{1} r_{1} + B^{-1} (r_{0}^{-1} p_{1} \ell_{3} - (c\sqrt {\gamma{^{{\prime\prime}}_{0}}})^{- 1} r_{1} \ell_{1} )]r_{1} \\ & \,\,\,\,\,\, + \varepsilon^{2} q_{1} \,\,[(c\,C\,\sqrt {\gamma{^{{\prime\prime}}_{0}} } \,)^{ - 1} (q_{1} \ell_{1} - p_{1} \ell_{2} ) + (x^{\prime}_{0} \gamma^{\prime}_{1} - y^{\prime}_{0} \gamma_{1} - C_{1} p_{1} q_{1} + kC_{1} \gamma_{1} \gamma^{\prime}_{1})] \\ & \,\,\,\,\,\, + A^{-1} \{r_{0}^{-1} [-\varepsilon \,b^{-1} (x^{\prime}_{0} \,\gamma{^{{\prime\prime}}_{1}} + kb\gamma_{1} \,\gamma{^{{\prime\prime}}_{1}} ) + \,p_{1} \,r_{1} + B^{ - 1} (r_{0}^{ - 1} p_{1} \ell_{3} - (c\sqrt {\gamma{^{{\prime\prime}}_{0}} } )^{ - 1} \,r_{1} \ell_{1} )]\,\ell_{3} \\ & \,\,\,\,\, - (c\sqrt {\gamma{^{{\prime\prime}}_{0}} } )^{ - 1} \,[\varepsilon^{2} ((cC\sqrt {\gamma{^{{\prime\prime}}_{0}} } )^{ - 1} (q_{1} \ell_{1} - p_{1} \ell_{2} ) + (x^{\prime}_{0} \gamma^{\prime}_{1} - y^{\prime}_{0} \gamma_{1} - C_{1} p_{1} q_{1} + kC_{1} \gamma_{1} \gamma^{\prime}_{1}))] \\ & \,\,\,\,\, \times \ell_{2}\} = \varepsilon \,a^{-1} \{ y^{\prime}_{0} \,(q_{1} \gamma_{1} - p_{1} \gamma{^{\prime}}) + k\,a[(\varepsilon p_{1} \gamma{^{{\prime\prime}}_{1}} - r_{1} \gamma_{1} )\,\gamma{^{{\prime\prime}}_{1}} + \,\gamma^{\prime}_{1} (q_{1} \gamma_{1} - p_{1} \gamma{^{\prime}})]\} . \\ \end{aligned}$$

The substitution from (5) to (7) of $r_{1}^{2} ,r_{1} \gamma{^{{\prime\prime}}_{1}} ,\gamma^{{\prime \prime 2}} ,$ and $\gamma{^{{\prime\prime}}_{1}}$ into the above equation yields the following first equation in the required system

8 $$\begin{aligned} \ddot{p}_{1} + \omega {{^{\prime 2}}} p_{1} =&\quad (cB\sqrt \gamma {{^{\prime\prime}_{0}} } )^{{ - 1}} \ell _{1} + \varepsilon \{ (Cr_{0} )^{{ - 1}} (q_{1} \ell _{1} - p_{1} \ell _{2} )q_{1} + (Br_{0} )^{{ - 1}} \ell _{1} S_{1} \\ & - \;(Ar_{0} )^{{-1}} \ell _{2} [C_{1} p_{1} q_{1} - (x^{\prime}_{0} + kC_{1} \gamma _{1} )\gamma ^{\prime}_{1} + y^{\prime}_{0} \gamma _{1} ] + b^{-1}x^{\prime}_{0} \\ & + \;(Ar_{0} )^{{-1}} (b^{{-1}} x{^{\prime}}_{0} + k\gamma _{1} )\ell_{3}\} + \varepsilon^{2}\{[b^{-1} x^{\prime}_{0} S_{2} + p_{1} (C_{1} q_{1}^{2} - \omega ^{2} S_{1} ) \\ & - \;q_{1} (x{{^{\prime}}_{0}} \gamma {{^{\prime}}_{1}} - y{{^{\prime}}_{0}} \gamma _{1} ) + a^{{ - 1}} y{{^{\prime}}_{0}} (q_{1} \gamma _{1} - p_{1} \gamma {{^{\prime}}_{1}} )] + kp_{1} (1 - \gamma {{^{\prime}}_{1}} ^{{ 2}} ) \\ & - \;\frac{1}{2}r_{0} ^{{ - 1}} \ell _{3} p_{1} (Ab)^{{ - 1}} [S_{1} - k(1 - \gamma {{^{\prime\prime}}_{1}}^{2} )] + (Ar_{0} )^{{ - 1}} (b^{{ - 1}} x{{^{\prime}}_{0} } + k\gamma _{1} )S_{2} \ell_{3} \}\\& + \;\frac{1}{2}\varepsilon^{3} \{ 4kS_{2} p_{1} - (Ar_{0} )^{{ - 1}} (k\gamma _{1} + b^{{ - 1}}x{^{\prime}}_{0})[S_{1} - k(1 - \gamma {{^{\prime\prime}}_{1}}^{2} )]\ell _{3} \} + \cdots . \\ \end{aligned}$$

In the same manner, one can derivative the fourth equation of system (4) and by using the other equations of the same system in addition to the substitution from (5)–(7) to obtain the following second equation of the desired system

9 $$\begin{aligned} \ddot{\gamma }_{1} + \gamma _{1} & = \left( {Br_{0} } \right)^{{ - 1}} \ell _{1} + \varepsilon \left[ {\left( {Br_{0} } \right)^{{ - 1}} \left( {\ell _{1} S_{2} - \ell _{3} p_{1} } \right) + \left( {Cr_{0} } \right)^{{ - 1}} \left( {q_{1} \ell _{1} - p_{1} \ell _{2} } \right)\gamma _{1}^{\prime } } \right] \\ & + \;\varepsilon ^{2} \left[ { - S_{1} \gamma _{1} + x_{0}^{\prime } \left( {\gamma _{1}^{{\prime 2}} + b^{{-1}}} \right) - \gamma _{1} \left( {y _{0}^{\prime } \gamma _{1}^{\prime } + q_{1}^{2} } \right) + k\left( {C_{1} \gamma _{1}^{{\prime 2}} + 1} \right)\gamma _{1}}\right] \\ & + \;\varepsilon ^{3} \left({2b^{{-1}} x_{0}^{\prime } + 2k\gamma _{1}} \right)S_{2} + \cdots , \\ \end{aligned}$$

where $\omega^{{\prime 2}} = 1 + \left( {\ell_{3} /Abr_{0} } \right)$ is known by Amer’s frequency^26,27,36^ which differs from the frequency $\omega$ by a samll amount depending on $\ell_{3}$.

Taking into account how $r_{0}$ is considered high. As a result, $r_{0}^{-2},r_{0}^{-3}, \ldots$ can be disregarded. The variables $q_{1}$ and $\gamma^{\prime}_{1}$ can be obtained from the system of Eqs. (4) and (7). They have the forms

10 $$\begin{aligned} & q_{1} = \,r_{1}^{ - 1} \,[1 - (A\,r_{0} \,r_{1} )^{ - 1} \,\ell_{3} + \; \cdots ][(c\,A\,\sqrt {\gamma{^{{\prime\prime}}_{0}} } \,)^{ - 1} \,r_{1} \,\ell_{2} - \dot{p}_{1} + \varepsilon \,a^{ - 1} (\,y^{\prime}_{0} \, + k\,a\,\,\gamma^{\prime}_{1})\,\gamma{^{{\prime\prime}}_{1}} ], \\ & \gamma^{\prime}_{1} = r_{1}^{-1} (\,\dot{\gamma }_{1} - \varepsilon \,q_{1} \,\gamma{^{{\prime\prime}}_{1}} \,). \\ \end{aligned}$$

New parameters $p_{2}$ and $\gamma_{2}$ can be introduced in such a way

11 $$p_{2} = p_{1} - \varepsilon \,\chi - \varepsilon \,\chi_{1} \,\gamma_{2} ,\quad \quad \gamma_{2} = \gamma_{1} - \varepsilon \, a\,p_{2} ,$$

where

$$\begin{aligned} & \chi = \frac{1}{{\omega^{{\prime 2}} }}\left[ {\frac{{x^{\prime}_{0}}}{b}\left( {1 + \frac{{\ell_{3} }}{{Ar_{0} }}} \right) + \frac{{y_{2}^{2} \ell_{1} }}{{Cr_{0} A^{2} }}} \right],\quad \quad \chi_{1} = - \frac{b}{{\ell_{3} }}(y^{\prime}_{0} \ell_{2} - k\ell_{3} ), \\ & y_{j} = (cC\sqrt {\gamma{^{{\prime\prime}}_{0}} } )^{ - 1} \ell_{j} ,\quad \quad (j = 1,2,3). \\ \end{aligned}$$

According to the new terms variables $p_{2}$ and $\gamma_{2}$, the variables $q_{1}$ and $\gamma^{\prime}_{1}$ can be estimated as follows

12 $$\begin{aligned} & q_{1} = - X\,(\,\dot{p}_{2} - a^{ - 1} y_{2} \,) + \varepsilon \,X\,[\;a^{ - 1} y^{\prime}_{0} - \chi_{2} \dot{\gamma }_{2} + (A\,r_{0} )^{ - 1} \,\ell_{2} \,S_{11} \;] + \varepsilon^{2} \,\{ \;X\,(k\,a + S_{11} )\dot{p}_{2} \\ & \,\,\,\,\,\,\,\,\,\, - \frac{1}{2}\, \,S_{11} \dot{p}_{2} + X\,(\,k\,\dot{\gamma }_{2} + a^{ - 1} y^{\prime}_{0}\,)\,S_{21} \;\} + \cdots , \\ & \gamma^{\prime}_{1} = \dot{\gamma }_{2} + X(A\,r_{0} )^{ - 1} \,\ell_{2} + \varepsilon \,[\;(a - X)\,\dot{p}_{2} + X\,(A\,r_{0} )^{ - 1} \,\ell_{2} \,S_{21} \;] + \varepsilon^{2} \{ \,X\,[\;a^{ - 1} y^{\prime}_{0} - \chi_{2} \dot{\gamma }_{2} \\ & \quad - S_{21} \dot{p}_{2} + (A\,r_{0} )^{-1}\,\ell_{2} \,S_{11} \;] - \frac{1}{2}\;S_{11} \,\dot{\gamma }_{2} \} + \cdots , \\ \end{aligned}$$

where

$$X = \,1 - \frac{{\ell_{3} }}{{A\,r_{0} }},\quad \chi_{2} = \chi_{1} - k\,,\quad \nu_{2} = (a - X).$$

Substituting (11) and (12) into (6), to obtain the next formulas of $S_{1}$ and $S_{2}$ based on the power series of $\varepsilon$

13 $$S_{i} = S_{{i 1}} + 2^{2 - i} \varepsilon \,S_{{i 2}} + \cdots ,\quad \quad \quad (\,i = 1,\;2\,)$$

where

14 $$\begin{aligned} & S_{11} = a\,(\,p_{20}^{2} - p_{2}^{2} \,) + b\,X^{2} (\,\dot{p}_{20}^{2} - \dot{p}_{2}^{2} \,) - 2\,x^{\prime}_{0} (\,\gamma_{20} - \gamma_{2} \,) - 2\,y^{\prime}_{0} (\,\dot{\gamma }_{20} - \dot{\gamma }_{2} \,) \\ & \quad \; + k\,[\;a\,(\,\gamma_{20}^{2} - \gamma_{2}^{2} \,) + b\,(\,\dot{\gamma }_{20}^{2} - \dot{\gamma }_{2}^{2} \;) + 2\,bX\,(A\,r_{0} )^{ - 1} \,(\dot{\gamma }_{20} - \dot{\gamma }_{2} )\,\ell_{2} \,] \\ & \quad \; - 2\,X^{2} BA^{ - 1} \,y_{2} \,(\dot{p}_{20} - \dot{p}_{2} \,), \\ & S_{12} = a\,[\,\chi \,(\,p_{20} - p_{2} \,) + \chi_{1} (\,p_{20} \,\gamma_{20} - p_{2} \,\gamma_{2} \,)\,] - b\,X^{2} \,\{ \;[\;(A\,r_{0} )^{ - 1} \ell_{2} \,S_{11} + a^{ - 1} \,y^{\prime}_{0}] \\ & \quad \; \times (\,\dot{p}_{20} - \dot{p}_{2} \,) - \chi_{2} (\,\dot{p}_{20} \,\dot{\gamma }_{20} - \dot{p}_{2} \,\dot{\gamma }_{2} \,)\;\} - x^{\prime}_{0} \,a\,(\,p_{20} - p_{2} \,) - y^{\prime}_{0} \,(a - X)(\,\dot{p}_{20} - \dot{p}_{2} \,) \\ & \quad \; - k\,S_{21} + k\,\{ \;a\,^{2} (\,p_{20} \,\gamma_{20} - p_{2} \,\gamma_{2} \,) + b\,(a - X)\,(\,\dot{p}_{20} \,\dot{\gamma }_{20} - \dot{p}_{2} \,\dot{\gamma }_{2} \,) + X(A\,r_{0} )^{ - 1} \\ & \quad \; \times b\,\ell_{2} [\;(\,\dot{\gamma }_{20} - \dot{\gamma }_{2} \,)\,S_{21} + (\,\dot{p}_{20} - \dot{p}_{2} \,)\,(a - X)\;]\;\} - X^{2} a^{ - 1} \chi_{2} \,y_{2} \,(\,\dot{\gamma }_{20} - \dot{\gamma }_{2} \,), \\ & S_{21} = a\,(\,p_{20} \,\gamma_{20} - p_{2} \,\gamma_{2} \,) - b\,X\,[\;(\,\dot{p}_{20} \,\dot{\gamma }_{20} - \dot{p}_{2} \,\dot{\gamma }_{2} \,) - X\,(A\,r_{0} )^{ - 1} \,\ell_{2} \,(\,\dot{p}_{20} - \dot{p}_{2} \,)\;] \\ & \quad \; + y_{1} \,(\,\gamma_{20} - \gamma_{2} \,) + y_{2} \,(\,1 - \,X\,B\,A^{ - 1} \,)(\,\dot{\gamma }_{20} - \dot{\gamma }_{2} \,), \\ & S_{22} = a\,[\;a\,(\,p_{20}^{2} - p_{2}^{2} \,) + \chi \,(\,\gamma_{20} - \gamma_{2} \,) + \chi_{1} (\,\gamma_{20}^{2} - \gamma_{2}^{2} \,)\;] + b\,X\{ \, - (a - X)(\dot{p}_{20}^{2} - \dot{p}_{2}^{2} \,) \\ & \quad \; + A^{ - 1} \,[\,C\,y_{2} \,(a - X) - X\,r_{0}^{ - 1} \,\ell_{2} \,S_{21} ](\,\dot{p}_{20} - \dot{p}_{2} \,) + [\ell_{2} \,(A\,r_{0} )^{ - 1} \,(\,S{}_{11} - X\,\chi_{2} \,) + a^{ - 1} y^{\prime}_{0}] \\ & \quad \; \times (\,\dot{\gamma }_{20} - \dot{\gamma }_{2} \,) - \chi_{2} (\,\dot{\gamma }_{20}^{2} - \dot{\gamma }_{2}^{2} \,)\;\} + y_{1} \,a\,(\,p_{20} - p_{2} \,) + y_{2} \,(\,\dot{p}_{20} - \dot{p}_{2} \,) - y_{3} \,S_{21} \;. \\ \end{aligned}$$

Inserting (13) and (14) into (7) to obtain

15 $$\begin{aligned} & r_{1} = 1 + \frac{1}{2}\, \varepsilon^{2} \,S_{11} + \varepsilon^{3} \,(S_{12} + kS_{21} ) + \cdots , \\ & \gamma{^{{\prime\prime}}_{1}} = 1 + \varepsilon \,S_{21} + \varepsilon^{2} \left( {S_{22} - \frac{1}{2}\, S_{11} } \right) - \varepsilon^{3} \,(S_{12} + k\,S_{21} ) + \cdots \;. \\ \end{aligned}$$

Utilizing (8) and (9), as well as (11)–(15), we are able to immediately produce the necessary system.

16 $$\begin{aligned} & \ddot{p}_{2} + \omega^{{\prime 2}} p_{2} = b^{ - 1} y_{1} + \varepsilon F(p_{2} ,\dot{p}_{2} ,\gamma_{2} ,\dot{\gamma }_{2} ,\varepsilon ), \\ & \ddot{\gamma }_{2} + \gamma_{2} = (Br_{0} )^{ - 1} \ell_{1} + \varepsilon \Phi (p_{2} ,\dot{p}_{2} ,\gamma_{2} ,\dot{\gamma }_{2} ,\varepsilon ), \\ \end{aligned}$$

where

$$F = F_{1} + \varepsilon \,F_{2} + \varepsilon^{2} \,F_{3} + \cdots ,\quad \quad \quad \quad \quad \quad \quad \Phi = \Phi_{1} + \varepsilon \,\Phi_{2} + \varepsilon^{2} \,\Phi_{3} + \cdots ,$$

$$\begin{gathered} F_{1} = - (B\,r_{0} )^{ - 1} \,\chi_{1} \,\ell_{1} + \,r_{0}^{ - 1} \,\ell_{2} \,\{ \;C^{ - 1} \,[\;(\,\dot{p}_{2}^{2} - 2\,A^{- 1} y_{2} \,\dot{p}_{2} ) - 2\,\dot{p}_{2} \,(\,a^{ - 1} y^{\prime}_{0} - \chi_{2} \,\dot{\gamma }_{2} \,)\;] \hfill \\ \quad \;\;\, + b\,A^{ - 1} p_{2} (\,\dot{p}_{2} - A^{ - 1} \,y_{2} \,)\;\} - (A\,r_{0} )^{ - 1} \,\ell_{2} \,[\;y^{\prime}_{0} \,\gamma_{2} + \dot{\gamma }_{2} \,(\,x^{\prime}_{0} + k\,C_{1} \,\gamma_{2} \,)\;], \hfill \\ \end{gathered}$$

$$\Phi_{1} = (B\,r_{0} )^{ - 1} \,S_{21} \,\ell_{1} + (C\,r_{0} )^{ - 1} \,\dot{\gamma }_{2} \,[\;A^{ - 1} \ell_{1} (\,A^{ - 1} y_{2} - \dot{p}_{2} \,) - \ell_{2} \,p_{2} \;]$$

$$\begin{gathered} F_{2} = f_{2} - \frac{{A^{2} r_{0} }}{{B\ell_{3} }}\,\chi_{1} p_{2} ,\quad \quad \quad \quad \quad \quad \quad \quad \Phi_{2} = \varphi_{2} + \frac{{A^{2} r_{0} }}{{B\ell_{3} }}\,(\,\chi + \chi_{1} \gamma_{2} \,), \hfill \\ F_{3} = f_{3} - \chi_{1} \,\varphi_{2} - \frac{{A^{2} r_{0} }}{{B\ell_{3} }}\,\chi_{1} \,(\,\chi + \chi_{1} \gamma_{2} \,),\quad \,\,\Phi_{3} = \varphi_{3} - a\,(\,f_{2} - \frac{{A^{2} r_{0} }}{{B\ell_{3} }}\chi_{1} p_{2} ), \hfill \\ \end{gathered}$$

$$\begin{aligned} & f_{2} = \,r_{0}^{ - 1} \ell_{2} \,\{ \;[\;2\,y_{2} (AC )^{ - 1} - p_{2} bA^{-1} ](\,a^{-1} y^{\prime}_{0} - \chi_{2} \dot{\gamma }_{2} ) - bA^{-1} (\chi + \chi_{1} \gamma_{2} ) \\ & \,\,\,\,\,\,\, \times (\,A^{ - 1} y_{2} - \dot{p}_{2} )\} + (C\,r_{0} )^{-1} \,\ell_{2} \,[\;(x^{\prime}_{0} + k\,C{}_{1}\,\gamma_{2} )\,\dot{p}_{2} + p_{2} \dot{\gamma }_{2} ] + 2\,(B\,r_{0} )^{ - 1} \ell_{1} S_{12} \\ & \quad - S_{11} p_{2} + \,x^{\prime}_{0}\,b^{-1} S_{21} + C_{{ 1}} X^{2} p_{2} \{ \;\dot{p}_{2}^{2} + (A^{-1} y_{2} - 2\,\dot{p}_{2}\,)A^{-1} y_{2} - 2\,\dot{p}_{2} [(A\,r_{0} )^{ - 1} \ell_{2} S_{11} \\ & \quad + a^{-1} y^{\prime}_{0} - \chi_{2} \dot{\gamma }_{2} ]\} + \{ \;y^{\prime}_{0} \,X\,(1 + a^{ - 1} )\,\gamma_{2} - x^{\prime}_{0} \,[X\,\dot{\gamma }_{2} + (A\,r_{0} )^{ - 1} \ell_{2} ]\} (A^{ - 1} y_{2} - \dot{p}_{2} ) \\ & \quad - a^{ - 1} p_{2} y^{\prime}_{0}\,\dot{\gamma }_{2} + \,k\,p_{2} \{ \;1 - \dot{\gamma }_{2}^{2} - 2\,(A\,r_{0} )^{ - 1} \,\ell_{2} \dot{\gamma }_{2} - 2\,[ \dot{\gamma }_{2} + (A\,r_{0} )^{ - 1} \ell_{2} ][\;(a - X) \dot{p}_{2} \\ & \quad + (A\,r_{0} )^{-1} \,\ell_{2} S_{21} ]\} + \frac{1}{2}\,\ell_{3} S_{11} p_{2} (Abr_{0})^{-1}+\chi_{1} \{ \;b^{-1} a y_{1} + r_{0}^{ - 1} \,[B^{ - 1} \ell_{1} S_{21} \\ & \,\,\,\,\,\, + C^{ - 1} \dot{\gamma }_{2} [ \ell_{1} (A^{-1} y_{2} - \dot{p}_{2} ) - \ell_{2} p_{2}]\,]\;\}, \\ \end{aligned}$$

$$\begin{aligned} & \varphi_{2} = r_{0}^{ - 1} \ell_{1} \{ \;B^{ - 1} S_{22} + C^{ - 1} [\;\nu_{2} \dot{p}_{2} (\,A^{ - 1} y_{2} - \dot{p}_{2} \,) + \dot{\gamma }_{2} (\,a^{-1} y^{\prime}_{0} - \chi_{2} \dot{\gamma }_{2} \,)\;]\;\} \\ & \quad - (C\,r_{0} )^{ - 1} \ell_{2} \,[\;p_{2} \dot{p}_{2} (a - X) + \dot{\gamma }_{2} (\,\chi + \chi_{1} \gamma_{2} \,)\;] - \gamma_{2} S_{11} + x^{\prime}_{0} \,\{ \;b^{ - 1} + \dot{\gamma }_{2} \,[\;\dot{\gamma }_{2} \\ & \quad + 2\,\ell_{2} (A r_{0} )^{ - 1} \,]\;\} - y^{\prime}_{0} \,\gamma_{2} \,[\;\dot{\gamma }_{2} + \ell_{2} (A r_{0} )^{ - 1}] + \gamma_{2} \{ k - X^{2} \,[\;\dot{p}_{2}^{2} \\ & \quad + A^{ - 1} y_{2} (\,A^{ - 1} \,y_{2} - 2\dot{p}_{2} \,)\;] + k\,C_{1} \,\dot{\gamma }\,[\;\dot{\gamma }_{2} + 2\,\ell_{2} (A \,r_{0} )^{ - 1} \;]\;\} \\ & \quad + 2S_{21} (x^{\prime}_{0}\, b^{-1} \, + \,k\,\gamma_{2}), \\ \end{aligned}$$

$$\begin{aligned} f_{3} = & \ell_{2} (A_{{ 1}} r_{0} )^{ - 1} \{ C^{ - 1} (a^{ - 1} y^{\prime}_{0} - \chi_{2} \dot{\gamma }_{2} )^{2} + [2C^{ - 1} (A^{ - 1} y_{2} - \dot{p}_{2} ) - p_{2} bA^{ - 1} \{ [k\nu \\ & + \;\frac{1}{2}S_{11} ]\dot{p}_{2} + (k\dot{\gamma }_{2} + a^{-1} y^{\prime}_{0}) S_{21} \} - bA^{ - 1} (\chi + \chi_{1} \gamma_{2} )(a^{ - 1} y^{\prime}_{0} - \chi_{2} \dot{\gamma }_{2} ) \\ & + \;a^{2} p_{2} \dot{p}_{2} + A^{ - 1} (x^{\prime}_{0} + kC_{1} \gamma_{2} )(a^{-1} y^{\prime}_{0} - \chi_{2} \dot{\gamma }_{2} - \dot{p}_{2} S_{21} )\} + b^{ - 1} x^{\prime}_{0}S_{22} \\ & - \;[2p_{2} S_{12} + S_{11} (\chi + \chi_{1} \gamma_{2})] + C_{1} p_{2} \{ 2A^{ - 1} X^{2} y_{2} [(Ar_{0} )^{ - 1} \ell_{2} S_{11} + a^{-1} y^{\prime}_{0} \\ & - \;\chi_{2} \dot{\gamma }_{2} ] - b\ell_{2} p_{2} (Ar_{0} )^{ - 1} (A^{ - 1} y_{2} - \dot{p}_{2} )\} + C_{1} X^{2} (\chi + \chi_{1} \gamma_{2} )\{ A^{ - 2} y_{2}^{2} \\ & + \;\dot{p}_{2} [\dot{p}_{2} - 2(A^{ - 1} y_{2} + a^{ - 1} y^{\prime}_{0} - \chi_{2} \dot{\gamma }_{2} - (Ar_{0} )^{ - 1} \ell_{2} S_{11} )]\} - Xx^{\prime}_{0}[(Ar_{0} )^{-1} \ell_{2} \\ & + \;\dot{\gamma }_{2} ][(Ar_{0} )^{ - 1} \ell_{2} S_{11} + a^{-1} y^{\prime}_{0} - \chi_{2} \dot{\gamma }_{2} ] + X(A^{ - 1} y_{2} - \dot{p}_{2} )\{ ap_{2} y^{\prime}_{0}(1 + a^{-1}) \\ & - \;x^{\prime}_{0}[a\dot{p}_{2} + (Ar_{0})^{-1} \ell_{2} S_{21} ]\} - a^{-1} y^{\prime}_{0} p_{2} (q_{1} + a \dot{p}_{2}) - (\chi + \chi_{1} \gamma_{2}) \\ & \times \;\{ a^{-1} y^{\prime}_{0} \dot{\gamma}_{2} - k [1 - \dot{\gamma }_{2}^{2} - 2(Ar_{0} )^{ - 1} \ell_{2} \dot{\gamma}_{2} - 2(\dot{\gamma }_{2} + (Ar_{0} )^{ - 1} \ell_{2} )[\dot{p}_{2} (a - X) \\ & + \;(Ar_{0} )^{ - 1} \ell_{2} S_{21} ]]\} + \frac{1}{2}\ell_{3} (Abr_{0} )^{-1} \{S_{11} (\chi + \chi_{1} \gamma_{2} ) + 2 p_{2} (S_{12} + S_{21} k)\} + \frac{1}{2}S_{11} \\ & \times \;[ - \ell_{3} (Ar_{0} )^{-1} (k \gamma_{2} + b^{ - 1} x^{\prime}_{0})] + 2p_{2} S_{21} k + a \chi_{1} \ell_{2} r_{0}^{ - 1} \{ \dot{p}_{2} C^{ - 1} [\dot{p}_{2} - 2(A^{-1} y_{2} \\ & + \;a^{ - 1} y^{\prime}_{0} - \chi_{2} \dot{\gamma }_{2} )] - b A^{ - 1} p_{2} (A^{ - 1} y_{2} - \dot{p}_{2} ) + A^{ - 1} [\dot{\gamma }_{2} (x^{\prime}_{0} + kC_{1} \gamma_{2} ) - y^{\prime}_{0} \gamma_{2} ]\} , \\ \end{aligned}$$

17 $$\begin{aligned} & \varphi_{3} = \ell_{1} \,(C\,r_{0} )^{ - 1} \{ (A^{ - 1} y_{2} - \dot{p}_{2} \,)(\,a^{-1} y^{\prime}_{0} - \chi_{2} \dot{\gamma }_{2} - S_{21} \dot{p}_{2} ) + \dot{\gamma }_{2} \,\left[ {\dot{p}_{2} \left( {k\, + \frac{1}{2}\;S_{11} } \right)} \right. \\ & \quad + (a^{ - 1} y^{\prime}_{0} + k\,\dot{\gamma}_{2} )\,S_{21} ] + \,\dot{p}_{2} (a - X)\,[\;S_{11} \ell_{2} (A\,r_{0} )^{ - 1} + a^{ - 1} y^{\prime}_{0} - \chi_{2} \dot{\gamma }_{2} ]\} - \ell_{2} \,(C\,r_{0} )^{ - 1} \\ & \quad \times [ p_{2} \,(\,a^{ - 1} y^{\prime}_{0} - \chi_{2} \dot{\gamma }_{2} - S_{21} \dot{p}_{2} ) + \nu_{2} \,\dot{p}_{2} (\chi + \chi_{1} \gamma_{2} \,)] - (\,a p_{2} S_{11} + 2\,\gamma_{2} \,S_{12} \,) \\ & \quad + 2\,x^{\prime}_{0} \,[\;\dot{p}_{2} (a - X) + \ell_{2} \,S_{21} \,(A\,r_{0} )^{ - 1} ][\dot{\gamma }_{2} + \ell_{2} (A\,r_{0} )^{ - 1} ] - y^{\prime}_{0}\,\{ \gamma_{2} [ \dot{p}_{2} (a - X) \\ & \quad + \ell_{2} \,S_{21} \,(A\,r_{0} )^{ - 1} ] + a\,p_{2} \,[\;\dot{\gamma }_{2} + \ell_{2} (A\,r_{0} )^{ - 1} ]\} - a\,p_{2} \,\{ X^{2} \,[\;\dot{p}_{2}^{2} + A^{ - 1} y_{2} (\,A^{ - 1} y_{2} \\ & \quad - 2\,\dot{p}_{2} )]\} - 2\,X^{2} \,\gamma_{2} (\,A^{ - 1} y_{2} - \dot{p}_{2} \,)[S_{11} \ell_{2} (A\,r_{0} )^{ - 1} + a^{ - 1} y^{\prime}_{0} - \chi_{2} \dot{\gamma }_{2} ] + 2\,k\,C_{1} \,\gamma_{2} [\dot{\gamma }_{2} \\ & \quad + \ell_{2} (A\,r_{0} )^{ - 1} ][ \dot{p}_{2} (a - X) + \ell_{2} \,S_{21} \,(A\,r_{0} )^{ - 1} ] + k\,a p_{2} \{ C_{1} \,\dot{\gamma }_{2} \,[\;\dot{\gamma }_{2} + 2\,\ell_{2} (A\,r_{0} )^{ - 1} ] + 1\,\} \\ & \quad + 2S_{21} (b^{ - 1} x^{\prime}_{0} + k \,\gamma_{2} )\;. \\ \end{aligned}$$

The integral of system (16) can be gained by using (5) as follows

18 $$\begin{aligned} & \gamma_{2}^{2} + \dot{\gamma }_{2} [\;\dot{\gamma }_{2} + 2\,\ell_{2} (A r_{0} )^{ - 1} \,] + 2\,\varepsilon \,\{ a\,\gamma_{2} p_{2} + [\;\dot{\gamma }_{2} + \ell_{2} (A r_{0} )^{ - 1} \,][\;\nu_{2} \dot{p}_{2} \\ & \;\;\;\; + \;\ell_{2} S_{21} (A r_{0} )^{ - 1} ] + S_{21} \} + \varepsilon^{2} \,\{ a^{2} p_{2}^{2} + \dot{p}_{2} (a - X)\,[\nu_{2} \dot{p}_{2} + 2\,\ell_{2} S_{21} (A\,r_{0} )^{ - 1} ] \\ & \;\;\;\; + \;2\,X\,[\;\dot{\gamma }_{2} + \ell_{2} (A r_{0} )^{ - 1} ][\ell_{2} \,S_{11} (A\,r_{0} )^{ - 1} + a^{ - 1} y^{\prime}_{0} - \chi_{2} \dot{\gamma }_{2} - S_{21} \dot{p}_{2} ] + S_{21}^{2} \\ & \;\;\;\;\left. { + \;2\,\left( {S_{22} - \frac{1}{2}\, S_{11} } \right)} \right\} = (\,\gamma{^{\prime\prime}}_{0} \,)^{-2} - 1\;. \\ \end{aligned}$$

Our goal is to determine whether this system has periodic solutions under the conditions $A > B > C$ or $A < B < C$ ($\omega^{{\prime 2}}$ is positive). In the first scenario, the body is placed in a quick starting spin $r_{0}$ around the major inertia ellipsoid axis, while in the second scenario; the body is positioned in a quick beginning spin $r_{0}$ around the minor inertia ellipsoid axis.

## Constructing the periodic solutions

This section's main objective is to find the periodic solutions of (16), taking into account the positive sign of $\omega^{{\prime 2}}$. It is obvious that the following conditions have no impact on the generality of the solutions $p,q,r,\gamma ,\gamma{^{\prime}},$ and $\gamma^{\prime \prime}$ due to the autonomously of system (16)

19 $$p_{2} (0,0) = b^{ - 1} y_{1} ,\quad \dot{p}_{2} (0,0) = 0,\quad \dot{\gamma }_{2} (0,\varepsilon ) = 0,$$

The generating system of (16) has the form

20 $$\ddot{p}_{2}^{(0)} + \omega^{{\prime 2}} p_{2}^{(0)} = 0,\quad \quad \ddot{\gamma }_{2}^{(0)} + \gamma_{2}^{(0)} = 0.$$

This system allows us the following periodic solutions with period $T_{0} = 2\pi \,n$

21 $$p_{2}^{(0)} = M_{1} \cos \,\omega{^{\prime}}\tau + M_{2} \sin \,\omega{^{\prime}}\tau ,\quad \gamma_{2}^{(0)} = M_{3} \cos \tau ,$$

where $M_{j} \;\;(j =1,2,3)$ indicate unknown constants can be determined later. In light of the foregoing, we can assume, the general desired periodic solutions of the autonomous system (16) has the form

22 $$\begin{aligned} & p_{2} (\tau ,\;\varepsilon ) = (\;M_{1} + \beta_{1} \,)\cos \omega{^{\prime}}\tau + (\,M_{2} + \beta_{2} \,)\sin \omega{^{\prime}}\,\tau + \sum\limits_{k = 1}^{\infty } {\varepsilon^{k} \,} G_{k} (\tau ), \\ & \gamma_{2} (\tau ,\;\varepsilon ) = (\;M_{3} + \beta_{3} \,)\cos \tau + \sum\limits_{k = 1}^{\infty } {\varepsilon^{k} \,} H_{k} (\tau );\,\,\,T(\varepsilon ) = T_{0} + \alpha (\varepsilon ) \\ \end{aligned}$$

where $T(\varepsilon )$ refers to the periodicity of the solutions. It's crucial to highlight that the constants $\beta_{1}$, $\omega{^{\prime}}\beta_{2}$ and $\beta_{3}$ reflect the differences between the initial values of $p_{2},\dot{p}_{2}$ and $\gamma_{2}$ of system (16) from their starting values of the system (20); these differences diminish when $\varepsilon = 0$. Through the following relations, one can provide the relevant conditions of these solutions (22) at $t = 0$.

23 $$\begin{aligned} & p_{2} (0,\varepsilon ) = M_{1} + \beta_{1} ,\quad \dot{p}_{2} (0,\varepsilon ) = \omega{^{\prime}}\,(\,M_{2} + \beta_{2} \,), \\ & \gamma_{2} (0,\varepsilon ) = M_{3} + \beta_{3} ,\quad \dot{\gamma }_{2} (0,\varepsilon ) = 0. \\ \end{aligned}$$

The following operator defines the functions $G_{k} (\tau )$ and $H_{k} (\tau )\;\;(k = 1,2,3, \cdots)$ in the context of^38^

24 $$U = u + \frac{{\partial u}}{{\partial M_{1} }}\,\beta_{1} + \frac{{\partial u}}{{\partial M_{2} }}\,\beta_{2} + \frac{{\partial u}}{{\partial M_{3} }}\,\beta_{3} + \frac{1}{2} \frac{{\partial^{2} u}}{{\partial M_{1}^{2} }}\,\beta_{1}^{2} + \cdots ,\quad \left( \begin{gathered} \,U = G_{k} ,\,H_{k} \, \hfill \\ \,u\,\; = g_{k} ,\;h_{k} \; \hfill \\ \end{gathered} \right)$$

where the expressions of the functions $g_{k} (\tau )$ and $h_{k} (\tau )$ can be defined mathematically as follows

25 $$\begin{aligned} & g_{k} (\tau ) = \frac{1}{{\omega{^{\prime}}}}\int\limits_{0}^{\tau } {F_{k}^{(0)} (t_{1} )\sin \,\omega{^{\prime}}(\tau - t_{1} )\,d t_{1} } , \\ & h_{k} (\tau ) = \int\limits_{0}^{\tau } {\Phi_{k}^{(0)} (t_{1} )\sin \,\omega{^{\prime}}(\tau - t_{1} )\,d t_{1} } \quad (k = 1,\;2,\;3)\;. \\ \end{aligned}$$

We are now looking for the determination of the functions $F_{1}^{(0)} ,\Phi_{1}^{(0)} ,F_{2}^{(0)} ,$ and $\Phi_{2}^{(0)}$. Therefore, solutions (21) must be reformulated in the following forms

26 $$\begin{aligned} & p_{2}^{(0)} = E\cos \,(\omega{^{\prime}}\tau - \eta ),\quad \quad \gamma_{2}^{(0)} = M_{3} \cos \tau ; \\ & E = \sqrt {M_{1}^{2} + M_{2}^{2} } ,\,\,\,\,\,\,\,\,\,\,\,\,\,\,\,\,\,\,\,\,\,\eta = \tan^{ - 1} M_{2} /M_{1} . \\ \end{aligned}$$

The substitution of (26) into (14) produces

$$\begin{aligned} S_{11}^{(0)} = & E^{2} \left[ {a\left( {\cos^{2} \eta - \frac{1}{2}} \right) + bX^{2} \omega^{{\prime 2}} \left( {\sin^{2} \eta - \frac{1}{2}} \right) + \frac{1}{2}(bX^{2} \omega^{{\prime 2}} - a)\cos 2(\omega{^{\prime}}\tau - \eta )} \right] \\ & - 2M_{3} [x^{\prime}_{0} (1 - \cos \tau ) + y^{\prime}_{0} \sin \tau ] - \frac{1}{2}kM_{3} [M_{3} C_{1} (1 - \cos 2\tau ) - 4b\ell_{3} (Ar_{0} )^{-1} \\ & \times \sin \tau ] - 2bE\omega{^{\prime}}X^{2} \ell_{2} (c A\sqrt {\gamma{^{{\prime\prime}}_{0}} } )^{ - 1} [\sin \eta + \sin (\omega{^{\prime}}\tau - \eta )], \\ \end{aligned}$$

$$\begin{gathered} S_{21}^{(0)} = M_{3} E\,\{ \,a\cos \eta \,\, + \frac{1}{2}(\,b\omega{^{\prime}}X - a\,)\,\cos \,[\,(\,\omega{^{\prime}} - 1\,)\tau - \eta \,] - \frac{1}{2}(\,b\omega{^{\prime}}X + a\,)\cos \,[(\,\omega{^{\prime}} + 1\,)\tau \hfill \\ \quad \,\,\,\, - \eta \,]\,\} + b X\,\{ \;a^{ - 1} M_{3} y_{2} \sin \tau - E\,\omega{^{\prime}}\,\ell_{2} (A\, r_{0} )^{ - 1} \,[\;\sin \eta + \sin \,(\,\omega{^{\prime}}\tau - \eta \,)\;]\;\} + M_{3} \hfill \\ \quad \,\,\,\, \times [\;y_{1} (\,1 - \cos \tau \,) + y_{2} \sin \tau \;], \hfill \\ \end{gathered}$$

27 $$\begin{aligned} & S_{12}^{(0)} = a\,E\;\{ X\;[\,\cos \eta - \cos \, (\,\omega{^{\prime}}\tau - \eta \,)\,] + \chi_{1} \,M_{3} [\,\cos \eta - \cos \tau \cos \;(\omega{^{\prime}}\tau - \eta )]\} - b\,X^{2} \,E\,\omega{^{\prime}} \\ & \quad \quad \times \{ [\ell_{2} S_{11}^{(0)} (A\,r_{0} )^{ - 1} + a^{ - 1} y^{\prime}_{0}\;][\sin \eta + \sin (\omega{^{\prime}}\tau - \eta )] + \chi_{2} \,M_{3} \sin \tau \sin (\omega{^{\prime}}\tau - \eta )\} - E\,\omega{^{\prime}} \\ & \quad \quad \times [a\,x^{\prime}_{0} + (a - X) y^{\prime}_{0}][\,\sin \eta + \sin (\omega{^{\prime}}\tau - \eta \,)\,] + k\,\{ a^{2} E\,M_{3} \,[\cos \eta - \cos \tau \cos (\omega{^{\prime}}\tau - \eta )] \\ & \quad \quad - b\,[\omega{^{\prime}} E M_{3} \,(a - X) \sin \tau \;\sin (\omega{^{\prime}}\tau - \eta \,) - \ell_{2} \,X\,(A\,r_{0} )^{ - 1} \,[M_{3} \,S_{21}^{(0)} \,\sin \tau + \omega{^{\prime}} E(a - X)\,(\sin \eta \\ & \quad \quad + \sin (\omega{^{\prime}}\tau - \eta ))]]\} - X^{2} M_{3} \,\chi_{2} \,\ell_{2} (\,c A\,\sqrt {\gamma{^{{\prime\prime}}_{0}} } \,)^{ - 1} \,\sin \tau - k\,\,S_{21}^{(0)} , \\ \end{aligned}$$

$$\begin{aligned} S_{22}^{(0)} = & a\{ aE^{2} [\cos^{2} \eta - \cos^{2} (\omega{^{\prime}}\tau - \eta )] + \chi M_{3} (1 - \cos \tau ) + \chi_{1} M_{3}^{2} \sin^{2} \tau \} \\ & + \;b\{ - E^{2} X\omega^{{\prime 2}} (a - X)[\sin^{2} \eta - \sin^{2} (\omega{^{\prime}}\tau - \eta )] + E\omega{^{\prime}}[y_{2} X(1 - aX) \\ & - \;\ell_{2} S_{21}^{(0)} (Ar_{0} )^{ - 1} ][\sin \eta + \sin (\omega{^{\prime}}\tau - \eta )] + M_{3} \sin \tau [X[\chi_{2} M_{3} \sin \tau \\ & + \;a^{ - 1} y^{\prime}_{0} + \ell_{2} S_{11}^{(0)} (Ar_{0} )^{ - 1} ] - \ell_{2} \chi_{2} (Ar_{0} )^{ - 1} ]\} + E\{ ay_{1} [\cos \eta - \cos (\omega{^{\prime}}\tau - \eta )] \\ & + \;\omega{^{\prime}} y_{2} [\sin \eta + \sin (\omega{^{\prime}}\tau - \eta )]\} - y_{3} S_{21}^{(0)} . \\ \end{aligned}$$

Making use of (26) and (27) into (17) to acquire the desired expressions of the functions $F_{1}^{(0)} ,\Phi_{1}^{(0)} ,F_{2}^{(0)} ,$ and $\Phi_{2}^{(0)}$ as follows

28 $$\begin{aligned} & F_{1}^{(0)} = 2\,\omega{^{\prime}}\,\ell_{2} \;(\,CA\,r_{0} \,)^{ - 1} (\,y_{2} + C\,y^{{\prime}_{0}} \,)(M_{1} \sin \omega{^{\prime}}\tau - M_{2} \,\cos \omega{^{\prime}}\tau ) + \cdots , \\ & \Phi_{1}^{(0)} = y_{2} \{ 1 + (CA)^{ - 1} (b\,X\, - \,r_{0}^{ - 1} \ell_{1} )\} \,M_{3} \sin \tau + \cdots , \\ & F_{2}^{(0)} = L(\omega{^{\prime}})\,(M_{1} \,\cos \omega{^{\prime}}\tau + M_{2} \,\sin \omega{^{\prime}}\tau ) + \cdots , \\ & \Phi_{2}^{(0)} = M_{3} \,N(\omega{^{\prime}})\cos \omega{^{\prime}}\tau + \cdots , \\ \end{aligned}$$

where

29 $$\begin{aligned} L(\omega{^{\prime}}) = & - \ell_{2} y^{\prime}_{0}b(aAr_{0})^{-1} - a\chi_{1} (1 - \omega^{{\prime 2}}) + [k + A^{-2} C_{1} (Xy_{2})^{2} - 2\ell_{1} a\chi (Br_{0})^{-1}] \\ & - \;\left[ {\frac{1}{2}(aBr_{0})^{-1} \ell_{3} + \omega^{2}} \right]\left\{ {\frac{1}{2} [a(2M_{1}^{2} - 1) + b \omega^{{\prime 2}} X^{2} (2M_{2}^{2} - 1)]} \right. \\ & - \;2M_{3} x^{\prime}_{0} - \frac{1}{2}[kM_{3}^{2} C_{1} + (b\omega^{{\prime 2}} X^{2} - a)(M_{1}^{2} + M_{2}^{2})] \\ & - \;2\ell_{2} b\omega{^{\prime}}M_{2} X^{2} (cA\sqrt {\gamma{^{{\prime\prime}}_{0}} } )^{ - 1} \} + \cdots , \\ \end{aligned}$$

$$\begin{aligned} N(\omega{^{\prime}}) = & - \;(aM_{1}^{2} + bX^{2} \omega^{{\prime 2}} M_{2}^{2} ) + X^{2} \omega^{{\prime 2}} (1 + b)(M_{1}^{2} + M_{2}^{2} ) + 2x^{\prime}_{0} M_{3} \\ & + \;a\chi_{1} (1 - \omega^{{\prime 2}} ) + k\left( {1 + \frac{1}{2}C_{1} M_{3}^{2} } \right) - (A^{ - 1} X y_{2} )^{2} - r_{0}^{ - 1} (\ell_{1} \chi a B^{ - 1} + \ell_{2} y^{\prime}_{0} A^{-1}) \\ & + \;2\ell_{2} b\omega{^{\prime}}M_{2} X^{2} (c A\sqrt {\gamma{^{{\prime\prime}}_{0}} } \,)^{ - 1} + 2k[M_{3} (aM_{1} + y_{1} ) - \ell_{2} b\omega{^{\prime}}M_{2} Xr_{0}^{ - 1} ] + \cdots . \\ \end{aligned}$$

Based on the formulas (25), (28) and (29), we can obtain directly the following expressions of $g_{1} (T_{0} ),g_{2} (T_{0} ),h_{1} (T_{0} ),h_{2} (T_{0} ),$ and their first derivatives

30 $$\begin{aligned} & g_{1} (T_{0} ) = 2\pi nM_{1} \ell_{2} \;(AC\,r_{0} )^{ - 1} (y_{2} + C\,y^{\prime}_{0}), \\ & \dot{g}_{1} (T_{0} ) = - 2\pi n\omega{^{\prime}}M_{2} \,\ell_{2} (AC\,r_{0} )^{ - 1} (\,y_{2} + C\,y^{\prime}_{0}), \\ & g_{2} (T_{0} ) = - \,\pi n(\omega{^{\prime}})^{ - 1} M_{2} \,L(\omega{^{\prime}}),\quad \dot{g}_{2} (T_{0} ) = \pi \,n\,M_{1} \,L(\omega{^{\prime}}), \\ & h_{1} (T_{0} ) = 0,\quad \quad \quad \quad \dot{h}_{1} (T_{0} ) = \pi nM_{3} \,y_{2} \{ 1 + A^{ - 1} \,[b\,X\,C - (C\,r_{0} )^{ - 1} \,\ell_{1} ]\} , \\ & h_{2} (T_{0} ) = 0,\quad \quad \quad \quad \dot{h}_{2} (T_{0} ) = \pi nM_{3} \,N(\omega{^{\prime}}\,). \\ \end{aligned}$$

The substitution of the conditions (23) into integral (18) at $\tau = 0$, produces

$$M_{3}^{2} + 2M_{3} \beta_{3} + \beta_{3}^{2} + 2\varepsilon [a(M_{1} + \beta_{1} )(M_{3} + \beta_{3} ) + \ell_{2} \omega{^{\prime}}(Ar_{0} )^{ - 1} (a - X)(M_{2} + \beta_{2} )] + \cdots = (\gamma{^{{\prime\prime}}_{0}} )^{ - 2} - 1.$$

If $\gamma{^{{\prime\prime}}_{0}}$ does not dependent on $\varepsilon ,$ we can acquire $M_{3}$ and $\beta_{3}$ as follows

31 $$M_{3} = (1 - \gamma{^{{\prime\prime}}_{0}}^{2} )^{{\tfrac{1}{2}}} (\gamma{^{{\prime\prime}}_{0}} )^{ - 1} \quad \quad 0 < M_{3} < \infty ,\quad \quad \beta_{3} = - \varepsilon a(M_{1} + \beta_{1} ) + \cdots .$$

The following provide the independent periodicity requirements can be expressed as follows ^22^

32 $$\begin{aligned} & - \pi n\beta_{2} (\omega{^{\prime}})^{ - 1} \{ L_{1} (\omega{^{\prime}}) - \omega^{{\prime 2}} N_{1} (\omega{^{\prime}})[1 + \ell_{1} (Br_{0} )^{ - 1} (M_{3} + \beta_{{ 3}} - \ell_{1} (Br_{0} )^{ - 1} )^{ - 1} ]\} + \varepsilon \, [\,G_{2} (T_{0} ) + \cdots \,] = 0, \\ & \pi n\beta_{1} \{ L_{1} (\omega{^{\prime}}) + N_{1} (\omega{^{\prime}})(b^{ - 1} y_{1} - \omega{^{\prime}}\beta_{{ 1}} )[1 + \ell_{1} (Br_{0} )^{ - 1} (M_{3} + \beta_{{ 3}} - \ell_{1} (Br_{0} )^{ - 1} )^{ - 1} ]\} + \varepsilon [\dot{G}_{2} (T_{0} ) + \cdots ] = 0, \\ & \varepsilon [M_{3} + \beta_{{ 3}} - \ell_{1} (B\,r_{0} )^{ - 1} ]^{ - 1} [\dot{H}_{1} (T_{0} ) + \varepsilon \dot{H}_{2} (T_{0} ) + \varepsilon^{2} \dot{H}_{3} (T_{0} ) + \cdots ] = \alpha (\varepsilon ). \\ \end{aligned}$$

The expressions of $L_{1} (\omega{^{\prime}})$ and $N_{1} (\omega{^{\prime}})$ can be formulated according to (32) by inserting $\beta_{{ 1}} ,\beta_{{ 2}} ,$ and $M_{3} + \beta_{{ 3}}$ instead of $M_{1} ,M_{2} ,$ and $M_{3}$, respectively. As a result, we may write

33 $$L_{1} (\omega{^{\prime}}) - \omega^{{\prime 2}} N_{1} (\omega{^{\prime}}) = (\beta_{1}^{2} + \beta_{{ 2}}^{2} )W_{1} (\omega{^{\prime}}) + kW_{2} (\omega{^{\prime}}) + W_{3} (\omega{^{\prime}}),$$

where

$$W_{1} (\omega{^{\prime}}) = \frac{1}{4} [b(\omega{^{\prime}} X)^{2} - a][2\omega^{{\prime 2}} - (aBr_{0} )^{ - 1} \ell_{3} ] - (1 + b)(\omega^{{\prime 2}} X)^{2} ,$$

$$W_{2} (\omega{^{\prime}}) = 1 - \frac{1}{4}C_{1} (aBr_{0} )^{ - 1} \ell_{3} (M_{3} + \beta_{3} )^{2} - \omega^{{\prime 2}} [1 + 2(a \beta_{1} + y_{1} )(M_{3} + \beta_{3} ) - 2\ell_{2} b\omega{^{\prime}}\beta_{2} (Ar_{0} )^{ - 1} ],$$

$$\begin{aligned} W_{3} (\omega{^{\prime}}) & = y^{\prime}_{0} \ell_{2} (r_{0} A)^{ - 1} [1 - (ab)^{ - 1} ] - a\chi \ell_{1} (Br_{0} )^{ - 1} + (Xy_{2} A^{ - 1} )^{2} (\omega^{{\prime 2}} + C_{1} ) \\ & - \;a\chi_{1} (1 - \omega^{{\prime}{4}} ) + \frac{1}{4}y_{1} \{ a + bX^{2} \omega^{{\prime 2}} + 4[x^{\prime}_{0} (M_{3} + \beta_{3} ) \\ & + \;\ell_{2} bX^{2} \omega{^{\prime}}\beta_{2} (cA\sqrt {\gamma{^{{\prime\prime}}_{0}} } )^{ - 1} ] + 2[a\beta_{1}^{2} + b(X\omega{^{\prime}}\beta_{2} )^{2} ]\} . \\ \end{aligned}$$

Since the *z*-axis must be oriented along either the minor or major axis of the body's inertia ellipsoid, then $W_{1} (\omega{^{\prime}}) > 0$ holds true for all $\omega{^{\prime}}$ that has been taken into account. Therefore, it is hypothesized that

$$k\, W_{2} (\omega{^{\prime}}) + W_{3} (\omega{^{\prime}}) \ne 0.$$

According to (32), each of $\beta_{{ 1}}$ and $\beta_{{ 2}}$ can be calculated as a power series of $\varepsilon$, in which they started with an order of $\varepsilon^{3}$. Therefore, the desired periodic solutions and the correction of the period $\alpha (\varepsilon )$ can be written as follows

$$p_{1} = \varepsilon \{ (\omega^{{\prime 2}} )^{ - 1} [x^{\prime}_{0} b^{ - 1} (1 + (Ar_{0} )^{ - 1} \ell_{3} ) + \ell_{1} y_{2}^{2} (CA^{2} r_{0} )^{ - 1} ] + \chi_{1} M_{3} \cos \tau \} + \cdots ,$$

$$\begin{aligned} q_{1} & = Xa^{ - 1} y_{2} - \varepsilon \{ \ell_{2} (Ar_{0} )^{ - 1} [2M_{3} [x^{\prime}_{0} (1 - \cos \tau ) + y^{\prime}_{0} \sin \tau ] + \frac{1}{2}kM_{3} [C_{1} M_{3} \\ & \times (1 - \cos 2\tau ) - 4b\ell_{3} (Ar_{0} )^{ - 1} \sin \tau ] - X(a^{ - 1} y^{\prime}_{0} + \chi_{2} M_{3} \sin \tau )\} + \cdots , \\ \end{aligned}$$

$$\begin{aligned} r_{1} & = 1 - \frac{1}{4}\;\varepsilon^{2} \,\{ \;4\,M_{3} \,[\;x^{\prime}_{0} \,(\,1 - \cos \tau \,) + y^{\prime}_{0} \sin \tau \,] + k\,M_{3} [\;C_{1} M_{3} \,(\,1 - \cos 2\tau \,) \\ & - 4\, b\,\ell_{3} \,(A\,r_{0} )^{ - 1} \sin \tau \,]\;\} + \; \cdots , \\ \end{aligned}$$

$$\gamma_{1} = M_{3} \cos \tau + \; \cdots ,$$

$$\begin{aligned} \gamma^{\prime}_{1} = & - M_{3} \sin \tau + \ell_{2} \,(A\,r_{0} )^{ - 1} \,\{ \;1 + \varepsilon \,M_{3} \,[\,y_{1} (\,1 - \cos \tau \,) + y_{2} \,\sin \tau \,]\;\} + \varepsilon^{2} \,\{ \;X\,(\,a^{ - 1} \,y^{\prime}_{0}\\ & + \;\chi_{2} \,M_{3} \sin \tau \,) - \frac{1}{2}\;[\;2\,\ell_{2} (A\,r_{0} )^{ - 1} + X\,M_{3} \sin \tau \,][\;2\,M_{3} \,[\,x^{\prime}_{0} \,(\,1 - \cos \tau \,) + y^{\prime}_{0} \sin \tau \,] \\ & + \;\frac{1}{2}\;k\,M_{3} \,[\;M_{3} \,C_{1} \,(\,1 - \cos 2\tau \,) - 4\,b\,\ell_{3} \,(A r_{0} )^{ - 1} \sin \tau \,]\;]\;\} + \; \cdots , \\ \end{aligned}$$

$$\begin{aligned} \gamma{^{{\prime\prime}}_{1}} = & 1 + \varepsilon \,M_{3} \,[\;y_{1} \,(\,1 - \cos \tau \,) + y_{2} \,\sin \tau - B\ell_{2} y_{2} (A^{2} r_{0} )^{ - 1} \sin \tau \;] + \varepsilon^{2} \,M_{3} \{ \;a\,\chi \\ & \times \;(\,1 - \cos \tau \,) + \frac{1}{2}\;M_{3} \,(\,a\,\chi_{1} + b\,X\,\chi_{2} \,)(\,1 - \cos 2\tau \,) + b\,[\,X a^{ - 1} y^{\prime}_{0} - \ell_{2} \chi_{2} \,(A\,r_{0} )^{ - 1} \,] \\ & \times \;\sin \tau - 2\,b\,\ell_{2} \,(A\,r_{0} )^{ - 1} \,[\;x^{\prime}_{0} \,(\,1 - \cos \tau \,) + y^{\prime}_{0} \sin \tau + \frac{1}{4}\;k\,M_{3} \,C_{1} \,(\,1 - \cos 2\tau \,)\;] \\ & \times \;M_{3} \sin \tau - y_{3} \,[\,y_{1} \,(\,1 - \cos \tau \,) + y_{2} \sin \tau - B\,\ell_{2} y_{2} (A^{2} \,r_{0} )^{ - 1} \sin \tau \;] + \,[\,x^{\prime}_{0} \,(\,1 - \cos \tau \,) \\ & + \,y^{\prime}_{0} \sin \tau + \frac{1}{4}\;k\,M_{3} \,C_{1} \,(\,1 - \cos 2\tau \,) - k\,b\,\ell_{3} \,(A\,r_{0} )^{ - 1} \sin \tau \;]\;\} + \; \cdots . \\ \end{aligned}$$

34 $$\begin{aligned} \alpha (\varepsilon ) & = - \,\varepsilon \pi n\{ 1 + \ell_{1} B^{ - 1} [r_{0} (M_{3} + \beta_{3} ) - \ell_{3} ]^{ - 1} \} [2x^{\prime}_{0} M_{3} - a\chi_{1} (1 - \omega^{{\prime 2}} ) + (y_{2} XA^{ - 1} )^{2} \\ & - \frac{1}{2}k(2 + C_{1} M_{3}^{2} ) + a\chi \ell_{1} (Br_{0} )^{ - 1} + y^{\prime}_{0} \ell_{2} (Ar_{0} )^{ - 1} - 2ky_{1} M_{3} ] + \cdots . \\ \end{aligned}$$

It is significant to note that the obtained solutions (40) do not involve any singular points owing to the usage of Amer’s frequency, unlike earlier works such as^22–24^ when $\omega$ equals $= 1,2,3, \cdots$ or their multiple inverses. The gained solutions are applicable for all rational values of $\omega{^{\prime}}$ and are considered generalizations of^33^ and^35^.

## Dynamical analysis of the body’s motion

This section's objective is to use Euler's angles (the angle nutation $\theta$, the angle of precession $\psi$, and the angle of spin $\varphi$) to visualize and analyse the disc's motion at any specific time. These angles are regarded as one of the most significant ways to depict how motion is oriented. The periodic solutions (34) nevertheless hold true if $t$ is changed to $t + t_{0}$, where $t_{0}$ is any arbitrary amount of time, because the initial system remains autonomous. Euler's angles have the following representations in terms of time $t$^1^

35 $$\cos \theta = \gamma^{\prime \prime},\quad \quad \frac{d\psi }{{d t}} = \frac{{p \gamma + q \gamma{^{\prime}}}}{{1 - \gamma^{{\prime \prime 2}} }},\quad \quad \tan \varphi_{0} = \frac{{\gamma_{0} }}{{\gamma^{{\prime}_{0}} }},\quad \quad \frac{d\varphi }{{d t}} = r - \frac{d\psi }{{d t}}\;\cos \theta .$$

Making use of (34) and (35), where $t + t_{0}$ is inserted instead of $t$, and then utilizing (4) to obtain the next formulas of $\theta ,\psi ,$ and $\varphi$

36 $$\begin{aligned} & \varphi_{0} = (\pi /2) + r_{0} h + \cdots ,\quad \quad \theta_{0} = \tan^{ - 1} M_{3} , \\ & \theta = \theta_{0} - \varepsilon \,\,[\;\theta_{{ 1}} (t + h) - \theta_{{ 1}} (h)\;] - \varepsilon^{{ 2}} \,\,[\;\theta_{{ 2}} (t + h) - \theta_{{ 2}} (h)\;], \\ & \psi = \psi_{0} + c\, \cos {\text{ec}} \theta_{0} \,\sqrt {\cos \theta_{0} } \;\{ \;[\psi_{1} (t + h) - \psi_{1} (h)\;] + \varepsilon \;[\;\psi_{2} (t + h) - \psi_{2} (h)] \\ & \;\;\; + \varepsilon^{2} \;[\;\psi_{3} (t + h) - \psi_{3} (h)\;]\;\} , \\ & \varphi = \varphi_{0} + r_{0} \,t - c\, \cot \theta_{0} \,\sqrt {\cos \theta_{0} } \;\{ [\varphi_{1} (t + h) - \varphi_{1} (h)\;] + \varepsilon \;[\varphi_{2} (t + h) - \varphi_{2} (h)]\} \\ & \quad - \varepsilon^{2} \;\{ \tan \theta_{0} \;[\varphi_{3} (t + h) - \varphi_{3} (h)] + c\, \cot \theta_{0} \,\sqrt {\cos \theta_{0} } \, [\varphi_{4} (t + h) - \varphi_{4} (h)]\} , \\ \end{aligned}$$

where

$$\theta_{1} (t) = - y_{1} \cos r_{0} t + [\;1 - c\, b\, \ell_{2} \,(A^{2} \,r_{0} )^{ - 1} \,]\;y_{2} \sin r_{0} t,$$

$$\begin{aligned} & \theta_{2} (t) = (\,y_{1} y_{3} - a\chi - x^{\prime}_{0} \,)\cos r_{0} \,t + \{ y^{\prime}_{0} \,(1 + a^{ - 1} bX\,) - y_{2} y_{3} - b\,(Ar_{0} )^{ - 1} [\ell_{2} ( \chi_{2} \\ & \quad \quad + \;2\,x^{\prime}_{0} \,\tan \theta_{0} + A\,^{ - 1} C_{1} y_{2} ) + k\,(\ell_{3} + \frac{3}{4}\,\ell_{2} C_{1} \,\tan^{2} \theta_{0} )]\} \sin r_{0} t + \frac{1}{2}\;\,\tan \theta_{0} \\ & \quad \quad \times \;[2\,b\,y^{\prime}_{0}\ell_{2} ( A\,r_{0} )^{ - 1} - (kC_{1} + a\,\chi_{1} + bX\chi_{2} )]\cos 2\,r_{0} t + b\, \ell_{2} \tan \theta_{0} \\ & \quad \quad \times \;(A\,r_{0} )^{ - 1} \,[x^{\prime}_{0}\sin 2\,r_{0} t + \frac{k}{4}C_{1} \tan\theta_{0} \sin 3\,r_{0} t], \\ \end{aligned}$$

$$\psi_{1} (t) = C\,y_{2} (A\,r_{0} )^{ - 1} \,[\;\ell_{2} \, A^{ - 1} t\,\,\cos \theta_{0} + \cos r_{0} t\;],$$

$$\begin{aligned} & \psi_{2} (t) = [( A\,r_{0} )^{ - 1} (2y^{\prime}_{0} \,\tan \theta_{0} + a\,^{ - 1} \,y_{1} y_{2} )\,\ell_{2} - \frac{1}{2}\;X\,\chi_{2} \,\tan \theta_{0} ]\,t \\ & \quad \quad \; + r_{0}^{ - 1} \,[\,x^{\prime}_{0}\,(\omega^{{\prime 2}} \,b)^{ - 1} \sin r_{0} t + \,a^{ - 1} \,y^{\prime}_{0} \cos r_{0} t + \frac{1}{4}\;\chi_{2} \,\tan \theta_{0} \sin 2 r_{0} t], \\ \end{aligned}$$

$$\begin{aligned} & \psi_{3} (t) = \frac{1}{2}\;\{ \;\chi_{1} \,\tan \theta_{0} + 2\,CX\,(A\,a )^{ - 1} y^{\prime}_{0} \,y_{2} \cot \theta_{0} - \,y_{2} \,\ell_{2} \,( aA\,r_{0} )^{ - 1} \,(\,4\,x^{\prime}_{0} + k\,C_{1} \,) \\ & \quad \quad \; + X^{2} a^{ - 1} y_{2} \tan \theta_{0} \,[k\,b\,\ell_{3} (A\,r_{0} )^{ - 1} - y^{\prime}_{0} \;] + \ell_{2} \,(A\,r_{0} )^{ - 1} \,(\,2\,a^{ - 1} \,y^{\prime}_{0}\,y_{1} + \chi_{2} \,y_{2} \\ & \quad \quad \; \times \tan \theta_{0} \;)\;\} \;t + \frac{1}{4}\;r_{0}^{ - 1} \tan \theta_{0} \,(y^{\prime}_{0} \,y_{2} a ^{ - 1} + \chi_{1} )\sin 2\,r_{0} t + C\,y_{2} \,( A\,r_{0} )^{ - 1} \\ & \quad \quad \; \times \left[ {\tan \theta_{0} \,\left( {x^{\prime}_{0} + \frac{1}{8}\;k\,C_{1} } \right) - \chi_{2} } \right]\, \cos r_{0} t - \frac{1}{24}y_{2} \,( a\,r_{0} )^{ - 1} \tan \theta_{0} \,[\;6\,x^{\prime}_{0} \;\cos 2\,r_{0} t \\ & \quad \quad \; + k\,C_{1} \cos 3\,r_{0} t\;], \\ \end{aligned}$$

$$\varphi_{1} (t) = \psi_{1} (t),\quad \quad \quad \varphi_{2} (t) = \psi_{2} (t),\quad \quad \quad \varphi_{4} (t) = \psi_{3} (t),$$

$$\begin{aligned} & \varphi_{3} (t) = x^{\prime}_{0} (\,r_{0} t - \sin r_{0} t) - y^{\prime}_{0} \cos r_{0} t + \frac{1}{8}\;k\,[C_{1} (2r_{0} t - \sin 2r_{0} t)\tan \theta_{0} \\ & \quad \quad \, + b\,\ell_{3} (A\,r_{0} )^{ - 1} \cos r_{0} t]\;. \\ \end{aligned}$$

where $\theta_{0} ,\psi_{0} ,\varphi_{0} ,$ and $r_{0}$ are arbitrary constants ($r_{0}$ is large). If they are chosen, it is possible to analyse the disc’s motion according to the obtained solutions (34). To be more specific, we note from the conditions $\gamma_{0} > 0,\quad 0 < \gamma{^{{\prime\prime}}_{0}} < 1$ that $\psi > 0$. Therefore speed of precession constant does not equal zero. Moreover, the comparison between the expressions (36) of Euler’s angles and the obtained ones in^22^ and^38^ shows consistency between them when the GMV vanishes, besides the absence of the Newtonian force field.

## Outcomes analysis

This section focuses on discussing the gained solutions (34) and the corresponding Euler’s angels (36) in light of their graphs. Therefore, let's have a look at the below data that affects body motion.

$$\begin{aligned} & A = 9\;{\text{kg}}\;{\text{m}}^{2} ,\;\;\;\;B = 2\;{\text{kg}}\;{\text{m}}^{2} ,\;\;\;\;R = 1000\;{\text{m}},\;\;\;\;r_{0} = 5\;{\text{rad}}\;{\text{s}}^{{ - 1}} , \\ & {\gamma{^{{\prime \prime }}} _{0}} = 0.001,\;\;\;\;M = 300\;{\text{kg}},\;\;\;\;{x^{\prime }}_{0} = 0.7\;{\text{m}},\;\;\;\;{y^{\prime }}_{0} = 0.3\;{\text{m}}, \\ & \ell_{j} ( = 50,\;100,\;150\,)\;{\text{kg}}\;{\text{m}}^{2} \;{\text{s}}^{ - 1}\;\;\;\;(j = 1,2,3) \end{aligned}$$

**Figure 2:**
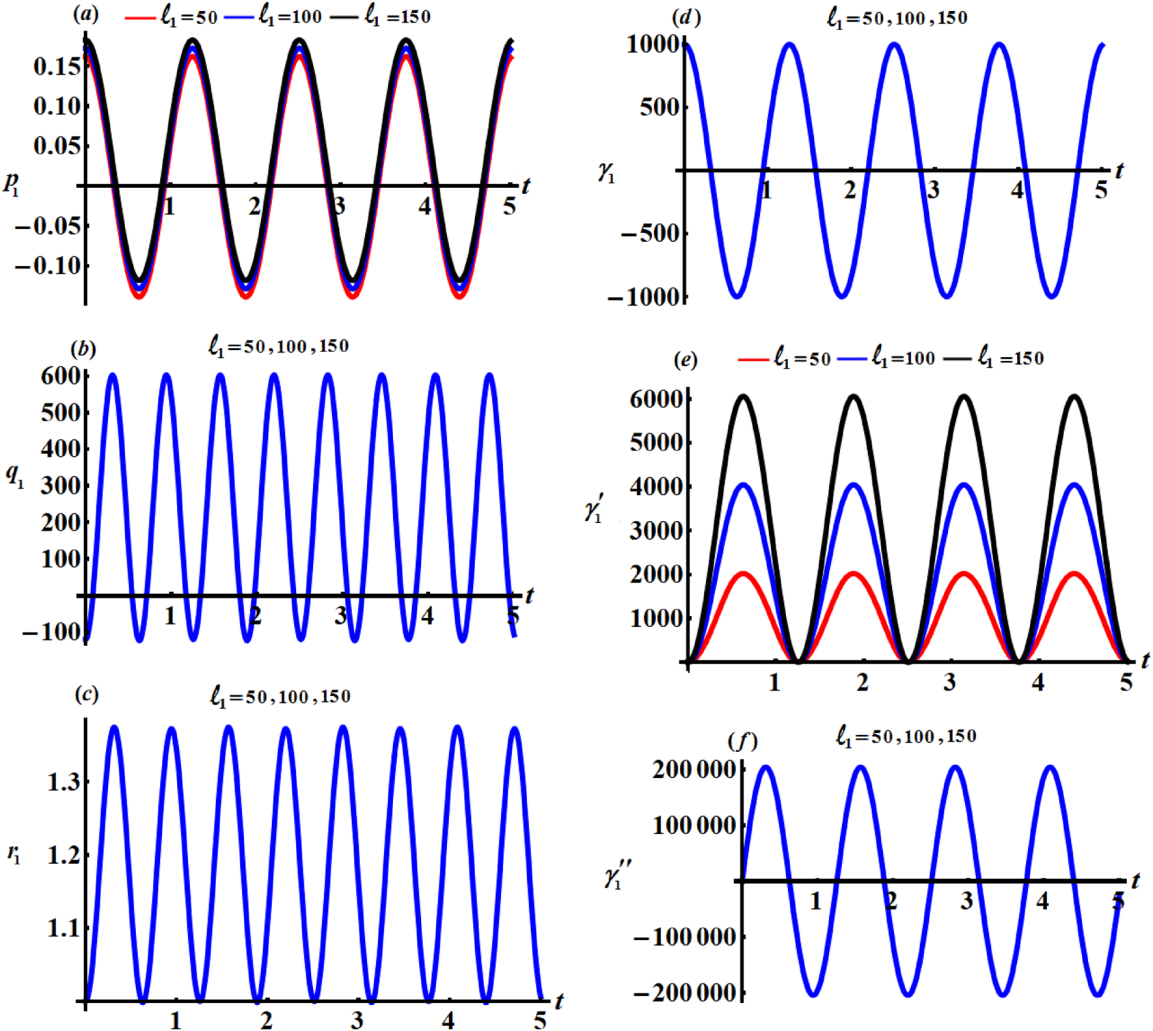
Represents the influence of $\ell_{1}$ on the solutions $p_{1} ,q_{1} ,r_{1} ,\gamma_{1} ,\gamma^{\prime}_{1},$ and $\gamma{^{{\prime\prime}}_{1}}$.

**Figure 3:**
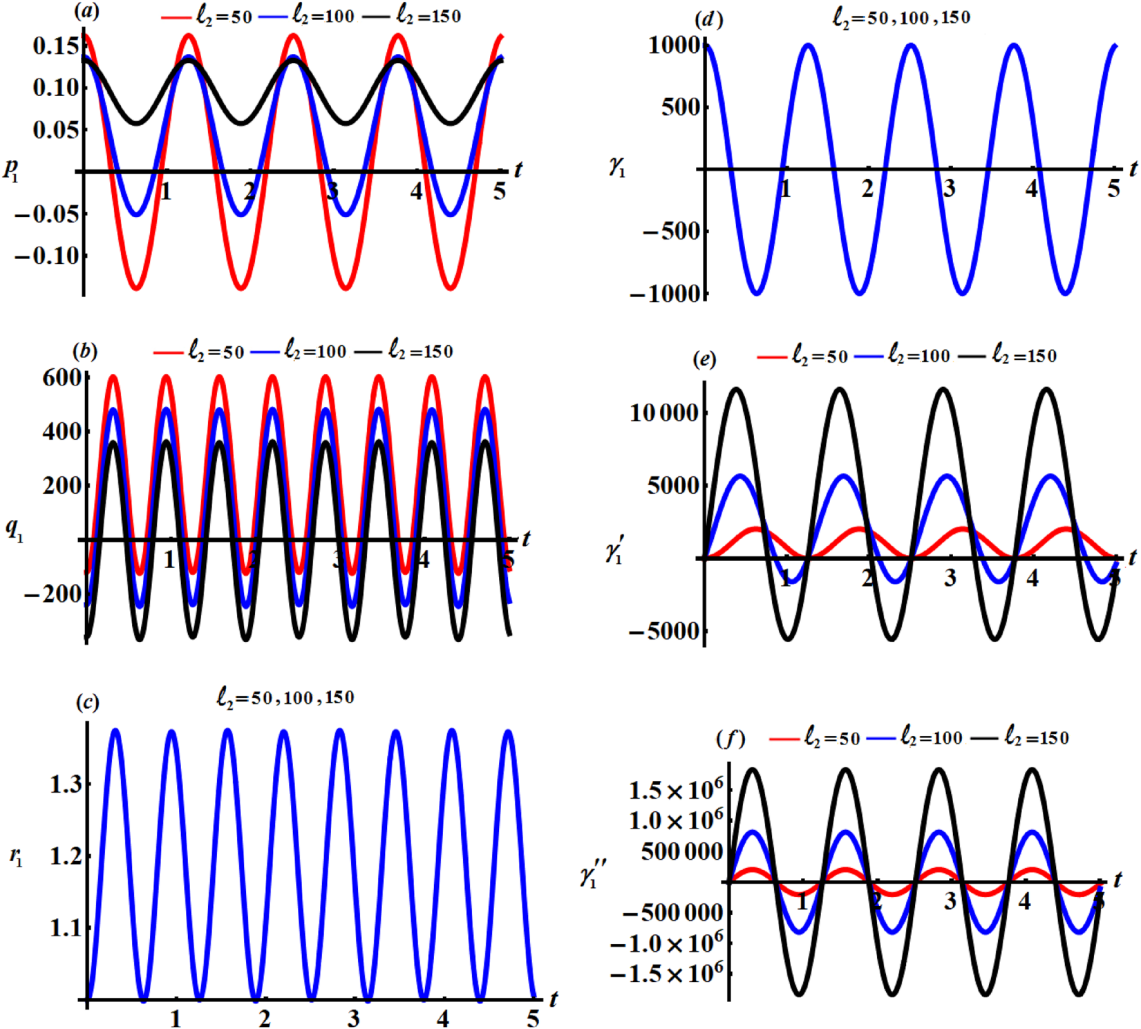
Describes the impact of $\ell_{2}$ on the solutions $p_{1} ,q_{1} ,r_{1} ,\gamma_{1} ,\gamma^{\prime}_{1} ,$ and $\gamma{^{{\prime\prime}}_{1}}$.

**Figure 4:**
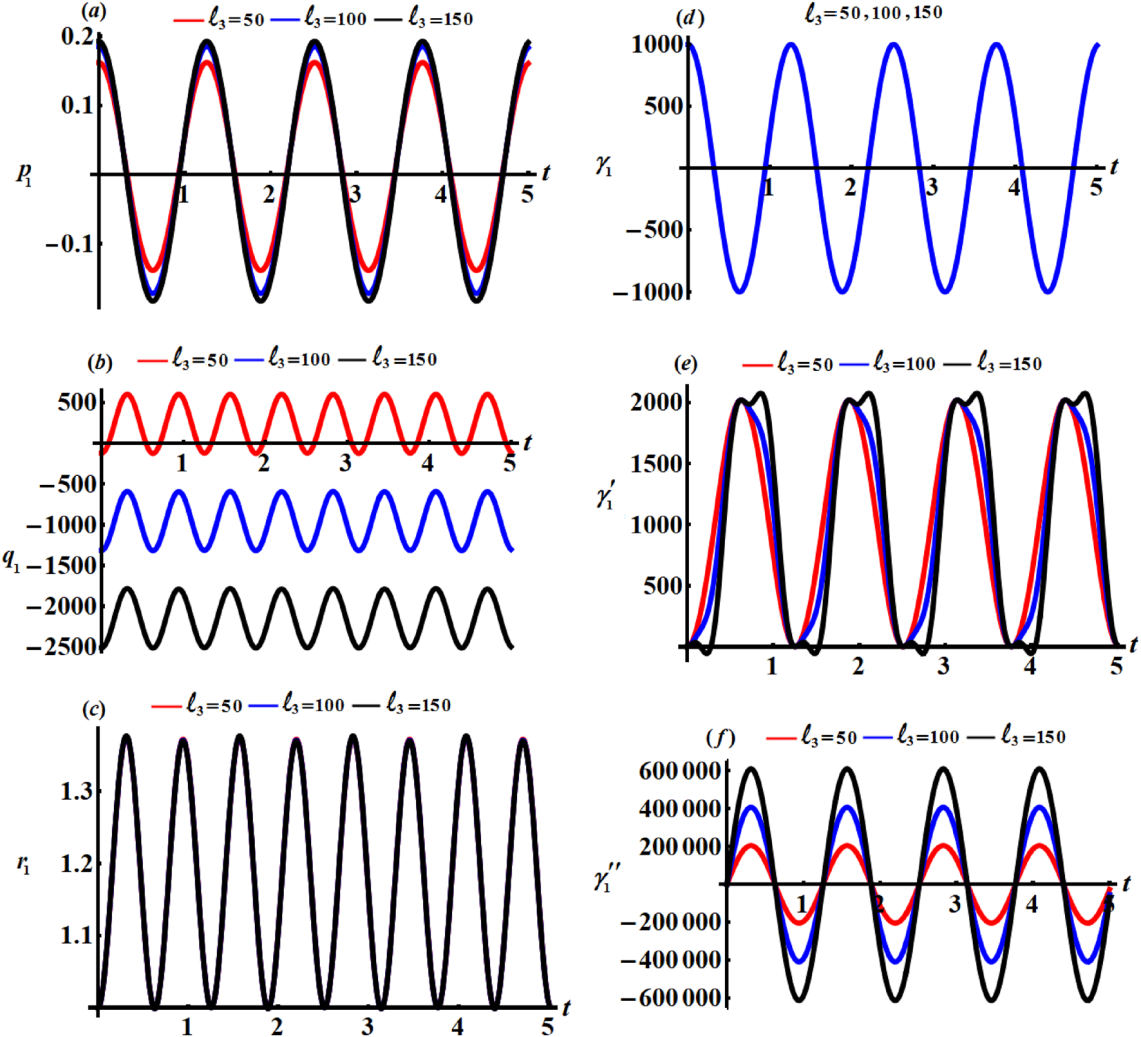
Shows the effects of $\ell_{3}$ on the gained solutions (34).

Figures 2, 3, and 4 show the influence of the projections of the GMV $\ell_{1} ,\ell_{2} ,$ and $\ell_{3}$ on the behaviour of the acquired solutions (34), respectively. These figures are drawn in matching of the above data. As expected before, the graphed solutions have periodic manner. The component $\ell_{1}$ had an excellent effect on the solutions $p_{1}$ and $\gamma_{1}^{\prime}$ as drawn in portions (*a*) and (*e*) of Fig. 2, while this impact becomes slightly with the other solutions as graphed in the other parts of Fig. 2. The reason is due to the mathematical structures of the achieved solutions (34). It is important to notice that the drawn waves in Fig. 2a and e are standing with some nodes, in which their amplitudes increase with the increase of $\ell_{1}$ values.

Looking more closely at the portions of Fig. 3 reveals that $\ell_{2}$ has a significant impact on the $p_{1} ,q_{1} ,\gamma^{\prime}_{1} ,$ and $\gamma{^{{\prime\prime}}_{1}}$ while this action can be neglected with the solutions $r_{1}$ and $\gamma_{1}$. The amplitudes of the periodic waves decrease with the increase of $\ell_{2}$ values as seen in Fig. 3a and b, in contrast we find that the amplitudes of the waves increase with an increment of $\ell_{2}$ values, as graphed in Fig. 3e and f. Additionally, one may also notice that the number of oscillations remains stationary.

One of the significant aspects is to study the influence of $\ell_{3}$ on the solutions $p_{1} ,q_{1} ,r_{1} ,\gamma_{1} ,\gamma^{\prime}_{1} ,$ and $\gamma{^{{\prime\prime}}_{1}}$. Therefore, curves of Fig. 4 are plotted to reveal its good action on the periodic behaviours of these solutions. The good effect of this component is evident with solutions $p_{1} ,q_{1} ,\gamma^{\prime}_{1} ,$ and $\gamma{^{{\prime\prime}}_{1}}$ as seen in Fig. 4a, b, e and f, while there is no significant effect with solutions $r_{1}$ and $\gamma_{1}$ as plotted in Fig. 4c and d.

**Figure 5:**
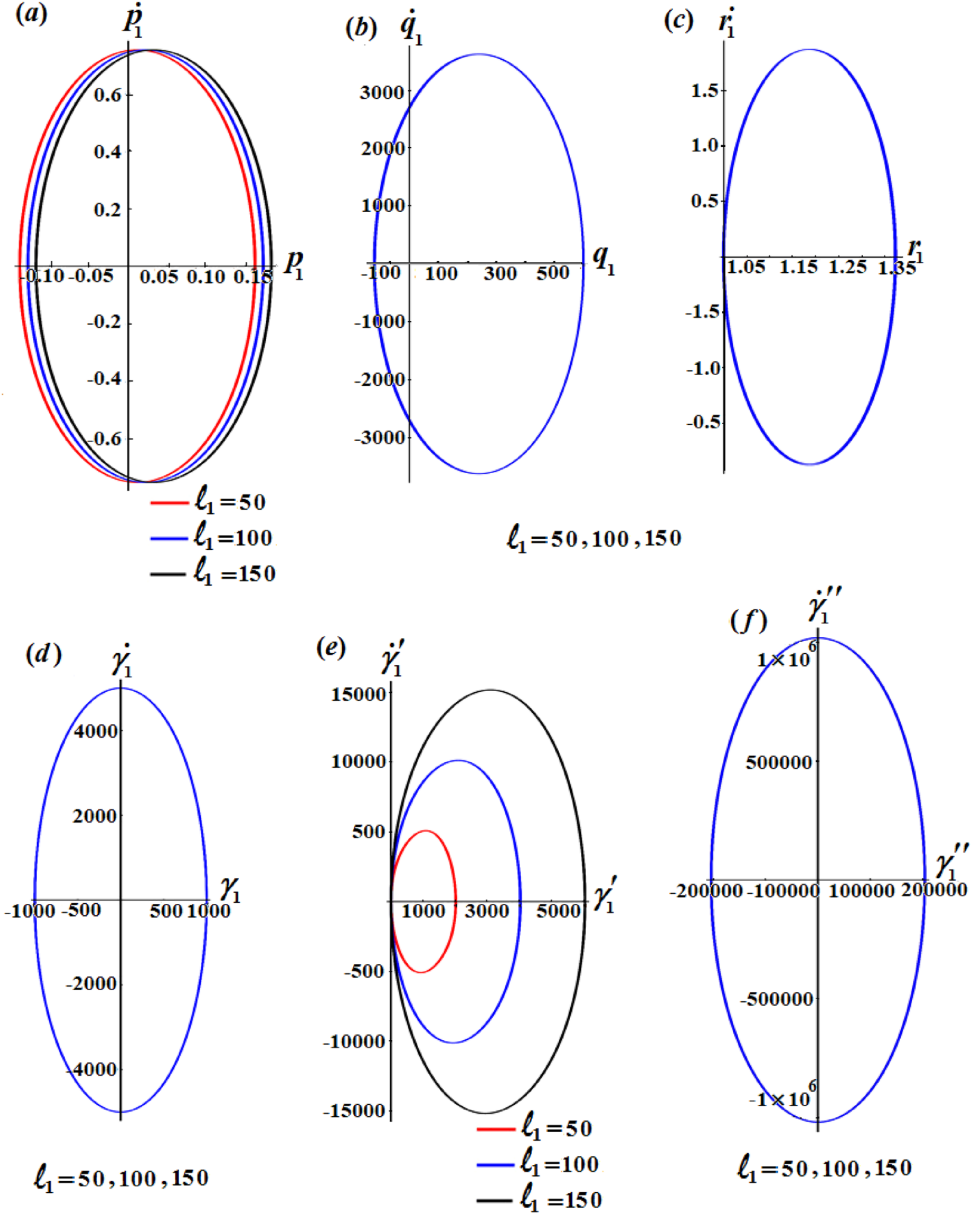
Reveals the phase plane plots of attained solutions at $\ell_{1} ( = 50,100,150)\;{\text{kg}}\;{\text{m}}^{2} \;{\text{s}}^{ - 1}$.

**Figure 6:**
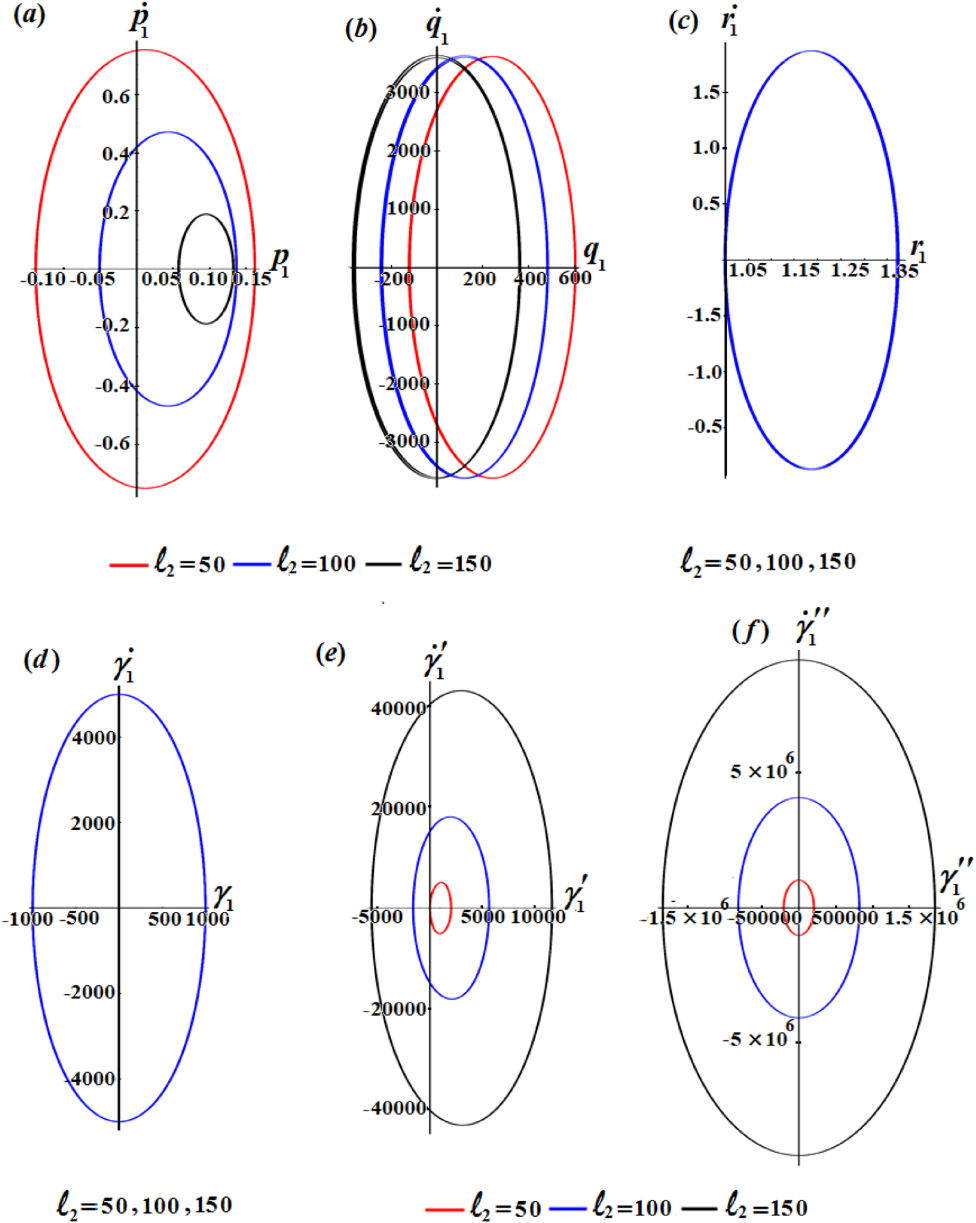
Shows the phase plane plots of attained solutions at $\ell_{2} ( = 50,100,150)\;{\text{kg}}\;{\text{m}}^{2} \;{\text{s}}^{ - 1}$.

**Figure 7:**
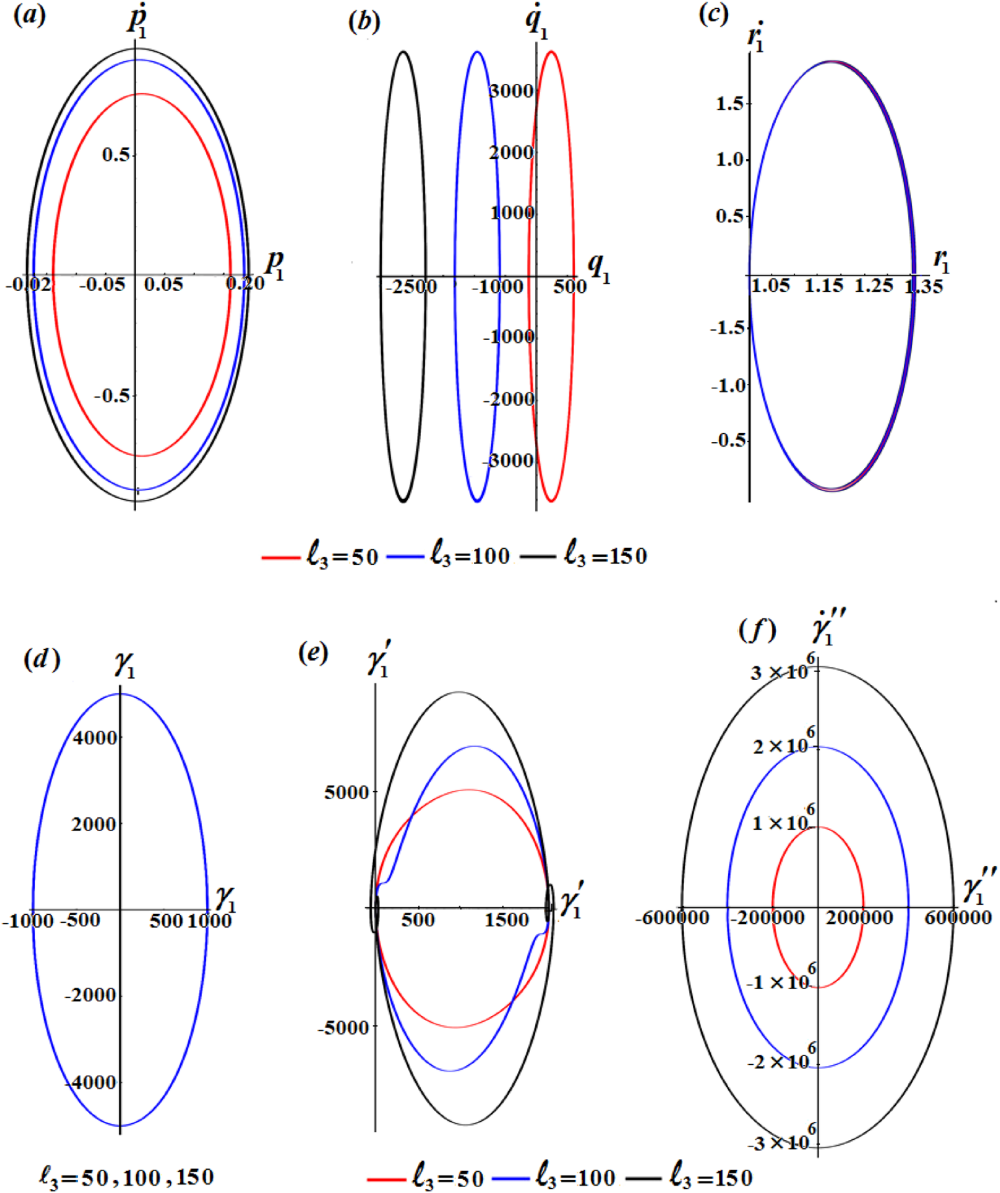
Explores the phase plane plots of attained solutions at $\ell_{3} (= 50,100,150)\;{\text{kg}}\;{\text{m}}^{2} \;{\text{s}}^{ - 1}$.

The phase plane diagrams of the solutions $p_{1} ,q_{1} ,r_{1} ,\gamma_{1} ,\gamma^{\prime}_{1} ,$ and $\gamma{^{{\prime\prime}}_{1}}$ are drawn in parts of Figs. 5, 6, and 7 for various values of $\ell_{1} ,\ell_{2} ,$ and $\ell_{3}$, respectively. These figures are created in the planes $p_{1} \dot{p}_{1} ,q_{1} \dot{q}_{1} ,r_{1} \dot{r}_{1} ,$ $\gamma_{1} \dot{\gamma }_{1} ,\gamma^{\prime}_{1} \dot{\gamma^{\prime}}_{1},$ and $\gamma{^{{\prime\prime}}_{1}} \dot{\gamma^{\prime \prime}}_{1}$. According to the periodicity of the solutions, the symmetric curves of these figures are closed, which denote the stability of the gained solutions. According to the variations of the solutions with the $\ell_{j}$ values, there is a change in the closed curves’ number, i.e., each periodic wave has only one corresponding closed curve.

**Figure 8:**
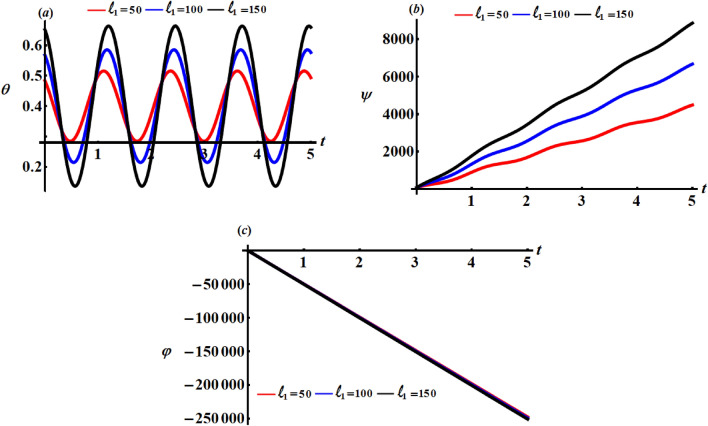
Describes the behaviour of the Euler’s angles at $\ell_{1} (= 50,100,150)\;{\text{kg}}\;{\text{m}}^{2} \;{\text{s}}^{ - 1}$.

**Figure 9:**
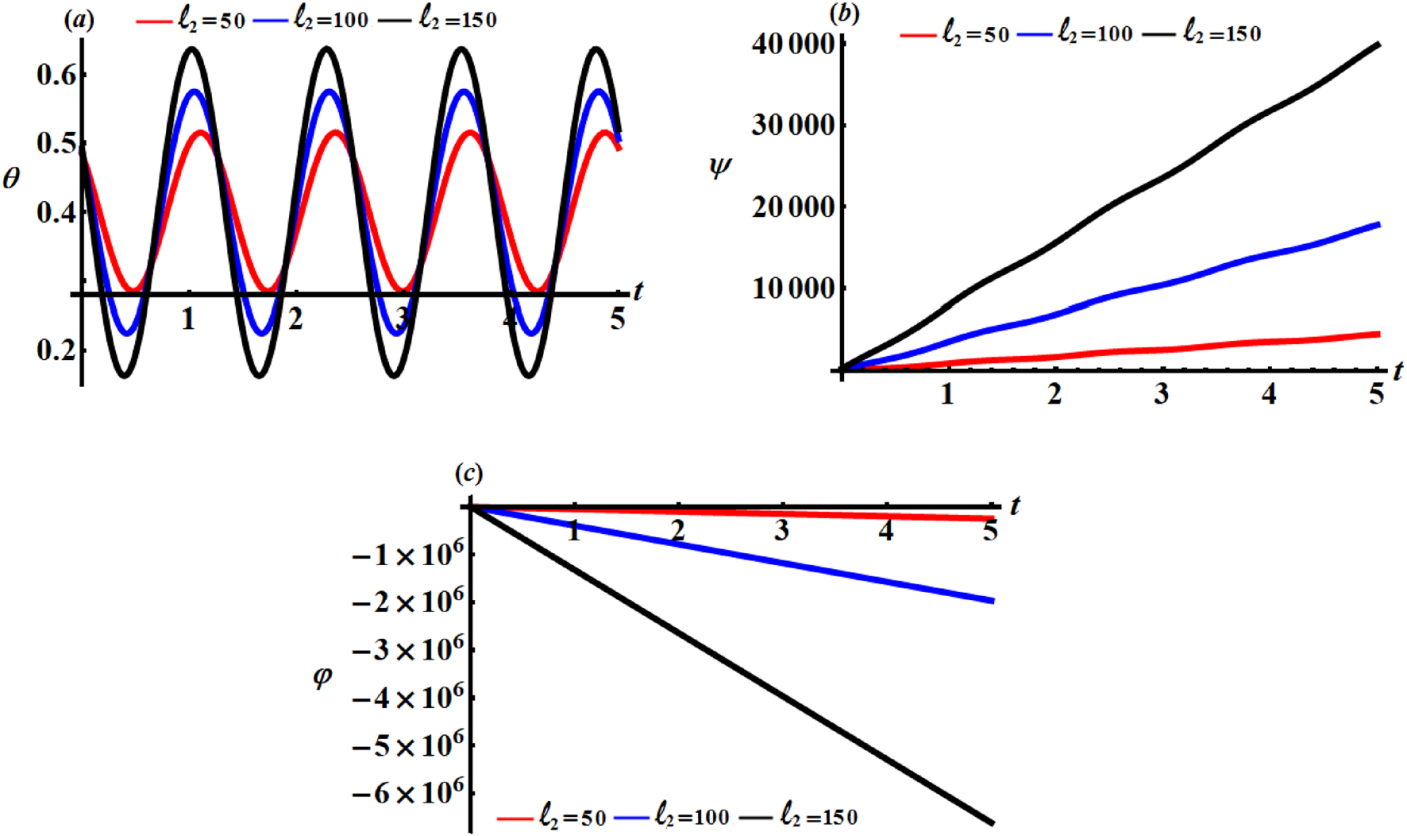
Describes the behaviour of the Euler’s angles at $\ell_{2} (= 50,100,150)\;{\text{kg}}\;{\text{m}}^{2} \;{\text{s}}^{ - 1}$.

**Figure 10:**
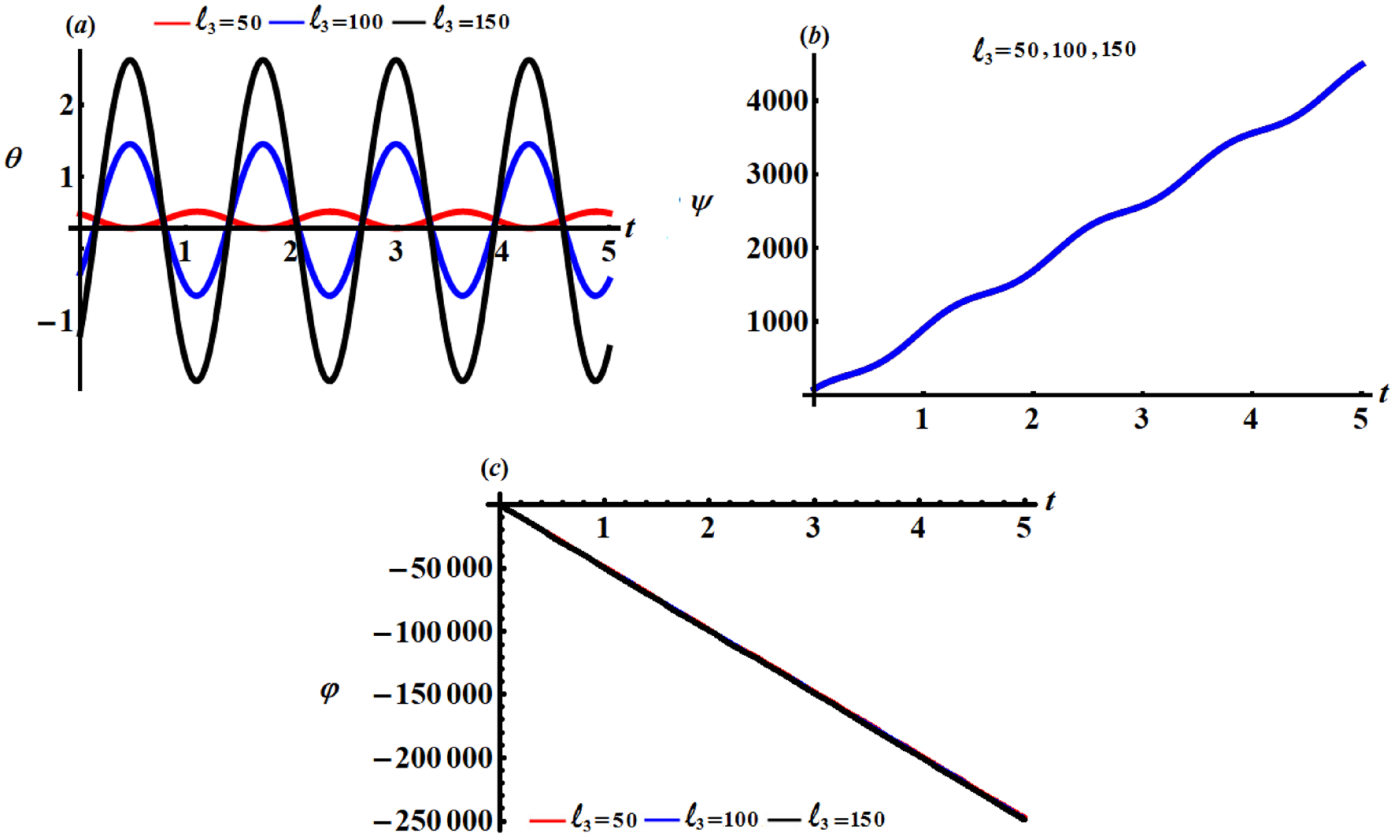
Describes the behaviour of the Euler’s angles at $\ell_{3} (= 50,100,150)\;{\text{kg}}\;{\text{m}}^{2} \;{\text{s}}^{-1}$.

Curves of Figs. 8, 9, and 10 characterize the variations of the angles $\theta ,\psi ,$ and $\varphi$ when $\ell_{j} (= 50,100,150);\,\,\,j = 1,2,3$. Examining these figures reveals that the angle $\theta$ has the periodicity forms with the change of $\ell_{j}$ as explored in parts (*a*) of Figs. 8, 9, and 10 i.e., it varies between increasing and decreasing, which is predicted from the $\theta$ equation of the system (36). Based on the curves of parts (*b*) and (*c*) of these figures, one can conclude that the time history of the angles $\psi$ and $\varphi$ increases and decreases with time, respectively. The reason is owing to the mathematical formulations of these angels as indicated in the system of Eqs. (36).

## Conclusion

The rotatory motion of a disc subjected to the NF and the GMV has been investigated. The controlling motion’s system in addition to their integrals, have been derived using the fundamental equation of angular momentum. In light of the PMSP, this system has been reduced to a quasi-linear autonomous one, besides only one integral, and then the approximate solutions and the Euler’s angles have been achieved. Along with the lack of the Newtonian force field, the comparison of the gained solutions and Euler's angles with the found ones of these works, which reveals consistency between them when the GMV vanishes. Singularities that typically show up in the solutions of previous treatments have been avoided by using the alternative frequency $\omega{^{\prime}}$ in place of $\omega$. In order to define how the body is oriented at any given time, Euler's angles as well as the obtained solutions are drawn to reveal the impact of the GMV on the disc’s motion. The solutions' stabilities have been assessed in accordance with the phase plane graphs of the solutions. The significance of this study may be traced to its wide range of applications in fields like physics, astronomy, and engineering dynamics applications, including assembly and machine design.

## Data Availability

The datasets used and/or analysed during the current study are available from the corresponding author on reasonable request.
